# Senescent Tumor Cells in the Peritoneal Carcinomatosis Drive Immunosenescence in the Tumor Microenvironment

**DOI:** 10.3389/fimmu.2022.908449

**Published:** 2022-06-30

**Authors:** Heidi Braumüller, Bernhard Mauerer, Christopher Berlin, Dorothea Plundrich, Patrick Marbach, Pierre Cauchy, Claudia Laessle, Esther Biesel, Philipp Anton Holzner, Rebecca Kesselring

**Affiliations:** ^1^ Department of General and Visceral Surgery, Medical Center – University of Freiburg, Faculty of Medicine, University of Freiburg, Freiburg, Germany; ^2^ German Cancer Consortium (DKTK) Partner Site Freiburg, Heidelberg, Germany; ^3^ German Cancer Research Center (DKFZ), Heidelberg, Germany

**Keywords:** Colorectal cancer, peritoneal carcinomatosis, tumor cell senescence, T cell senescence, stem cell phenotype

## Abstract

More than half of all patients with colorectal cancer (CRC) develop distant metastasis and, depending on the local stage of the primary tumor, up to 48% of patients present peritoneal carcinomatosis (PC). PC is often considered as a widespread metastatic disease, which is almost resistant to current systemic therapies like chemotherapeutic and immunotherapeutic regimens. Here we could show that tumor cells of PC besides being senescent also exhibit stem cell features. To investigate these surprising findings in more detail, we established a murine model based on tumor organoids that resembles the clinical setting. In this murine orthotopic transplantation model for peritoneal carcinomatosis, we could show that the metastatic site in the peritoneum is responsible for senescence and stemness induction in tumor cells and that induction of senescence is not due to oncogene activation or therapy. In both mouse and human PC, senescence is associated with a senescence-associated secretory phenotype (SASP) influencing the tumor microenvironment (TME) of PC. SASP factors are able to induce a senescence phenotype in neighbouring cells. Here we could show that SASP leads to enhanced immunosenescence in the TME of PC. Our results provide a new immunoescape mechanism in PC explaining the resistance of PC to known chemo- and immunotherapeutic approaches. Therefore, senolytic approaches may represent a novel roadmap to target this terminal stage of CRC.

## Introduction

Colorectal cancer (CRC) is the second leading cause of cancer-related deaths in the Western world (1). Although there are increasing efforts for better clinical and mechanistic insights, CRC patients displaying metastasis at the time of diagnosis show a poor five-year survival rate of only 14% (1). In this context, patients with PC show worst prognosis (2) and do not benefit from systemic chemotherapy. The only successful therapy so far is the removal of all visible metastases (3). One possible reason for the resistance of PC patients to chemotherapy is the low penetration of systemically applied chemotherapeutics in the peritoneal cavity (4). To overcome this resistance, Sugarbaker introduced a heated intraperitoneal chemotherapy (HIPEC) nearly 30 years ago (5). However, the advantage of HIPEC together with cytoreductive surgery is not clear as a (clinical) phase III trial showed no superior overall survival in the cytoreductive surgery (CRS) plus HIPEC group as compared with cytoreductive surgery alone (6). Senescence, a permanent cell cycle arrest, has long been viewed as a mechanism against malignant transformation (7). In a mouse model recapitulating pancreatic cancer development, the prevention of senescence leads to excessive tumor growth, highlighting the need for senescence induction in cancer containment (8, 9). In general, stressed cells halt their cell cycle machinery and become either apoptotic or become senescent. We previously showed that PC is characterized by tumor cells showing features of senescence (10). These results argue against the long held view that senescence amounts to permanent cell-cycle arrest that prevents cancer formation and progression in mammals. In line with human PC samples, several studies could show that senescent cells can escape from senescence and give rise to aggressive tumors in human cell lines and in murine cancer models (11–13).

Although senescent cells are arrested in the G1 or G2/M phase of the cell cycle (14, 15), they heavily upregulate proinflammatory molecules encompassed by SASP *via* active metabolic reprogramming (16). Secretion of proinflammatory molecules mediate many of the physiopathological effects of senescent cells.Although senescent PC cancer cells should attract immune cells and get eliminated through the immune surveillance (17, 18), most PC cancer cells upregulate senescence markers and accumulate within the tumor. Senescent cancer cells that escape from growth arrest and resume proliferation appear to express stem cell markers, linking senescence-associated stemness with treatment failure and subsequent relapse (19). Senescent cells are able to induce a senescent phenotype in adjacent cells, including immune cells (20). Yet, to date, the role of senescence in the tumor microenvironment (TME) remains poorly characterized.

*In vivo* mouse models of CRC develop a tumor burden that causes death of the animals before solid tumors can even progress to a metastatic stage (21–23). Fumagalli et al. developed an orthotopic transplantation model of 3D epithelial organoid cultures, which not only progress to a primary solid tumor but also form distant metastases in the liver and the lung (24, 25). These organoids thus mimic primary tumors and are therefore suitable to recapitulate the clinical situation precisely (26). Therefore, we adapted this model for PC to analyze the cancer cells and the TME in (more) detail, and to compare PC with both primary tumor and liver metastasis samples.

We previously described a senescence phenotype within tumor cells of peritoneal carcinomatosis using human patient data. In the present study, we show, in patient specimens and in a mouse model, that senescent tumor cells are associated with increased stemness as seen by the upregulation of stem cell markers. Using our murine transplantation model, we demonstrate that PC samples similarly upregulate senescence and stem cell markers as found in human PC. In comparison to primary tumors, murine liver metastases showed no upregulation of these markers, indicating that the site of metastasis by itself, rather than oncogene expression or therapy, is critical for senescence. Both human and murine senescent PC cells showed unique SASP features, with factors that are known to induce senescence in neighboring cells. Our results show that tumor-infiltrating lymphocytes (TILs), derived from human and murine PC, carry features of dysfunctionality such as PD-1 and senescence such as IFN-γ or Tim-3 and conversely downregulate co-stimulatory molecules such as CD27 or CD28. Induction of immunosenescence by senescent tumor cells is not limited to PC but could be a broader escape mechanism of aggressive tumors that are resistant to checkpoint inhibitor blockade. within the most aggressive malignant primary brain tumor, the Glioblastoma multiforme (GBM), several studies could show that patients with GBM have significantly more senescent cancer cells and more senescent T cells not only in the TME but also systemically in the peripheral blood compared with healthy age matched controls (27–29). Our results provide insights into a unique metastatic tumor site and shed new light on the mechanistics of senescence and immunosenescence within PC. Senescent PC cells harboring stem cell characteristics and senescent TILs could thus account for resistance to chemotherapy and immunotherapy, repectively.

## Material and Methods

### Human Samples

Patients suffering from PC or liver metastasis caused by cancer of the appendix, colon, rectosigmoid or rectum as primary tumor sites were included. For immunohistochemistry, FFPE samples of patients (n = 50) who had tumor surgery on PC of colorectal origin between 2004 and 2019 were analyzed. The mean peritoneal cancer index (PCI), measuring PC extent (total score 1–39) of all patients was 16. The clinicopathological characteristics are summarized in the **Supplementary Table 2**. The extraction of samples was conducted by experienced surgeons. Tumor tissue for FACS analysis was excised during routine pathological examination. FACS analysis was performed with samples of patients (primary colorectal cancer CRC n=5, liver metastases n=5, PC n=5) treated with tumor surgery in 2021. PC patients’ records in the computer database were analyzed with regard to pathological diagnosis, TNM staging and to obtain patient survival. All patients gave written consent. This study was conducted according to the Declaration of Helsinki and was approved by the ethics committee of the University of Regensburg and University of Freiburg.

### Mice

C57BL/6J mice were provided by Charles River (Sulzfeld, Germany). Animal experiments were in accordance with animal welfare regulations and had been approved by the local authorities (Regierungspräsidium Freiburg).

### Organoids and Organoid Culture

APTAK organoids are tumor organoids that are devoid of Apc, Tp53, Tgfbr2 and express constitutively active Kras (Kras^G12D^) and an activated/myristoilated isoform of Akt1. The organoids are a gift from F. Greten, (Georg-Speyer-Haus, Frankfurt).

The APTAK organoids were isolated from a colon of a p53^flox/flox^::Tgfbr2^flox/flox^ mouse. The established organoids harbor loxP sites flanking exon4 of the TGFBR2 gene and Intron 1 and 10 of the TP53 gene. The APC knock out was introduced using CRISPR/Cas9 transgenesis and confirmed by Western Blotting. The organoids were transfected with Cre-IRES-puroR plasmid (Addgene #30205) that encode Cre recombinase to ablate Tgfbr2 and p53. Kras gain of function was obtained by overexpressing the constitutive active version of the murine KRAS gene KRAS^G12D^ (cloned in Addgene plasmid #111164) by retroviral transduction. Myristoilated Akt1 was obtained by overexpression the active form of Akt1 (cloned in Addgene plasmid #) by retroviral transduction. APTAK organoids were cultured in basal medium (Advanced DMEM-F12 supplemented with penicillin/streptomycin, HEPES (10mmol/L, Invitrogen), Glutamax 1x (Invitrogen), N2 1x (Gibco), B27 1x (Gibco), and N-Acetylcysteine (1mmol/L, Sigma-Aldrich). Hygromycin (200 µg/mL) and puromycin (2 µg/mL) was added to maintain that the genes are mutated. The organoids were subcultured in Cultrex Reduced Growth Factor Basement Membrane Extract, Type 2 (R&D Systems, Bio-Techne, USA) and passaged every 5-7 days.

### Surgical Transplantation of Organoids Under the Subserosa of the Cecum

In Cultrex Reduced Growth Factor Basement Membrane Extract grown APTAK organoids were dissociated into a single-cell suspension by mechanical disruption followed by enzymatic digestion for 20 minutes at 37°C using TrypLE (Gibco) and were washed once in ice-cold PBS. Single cells were then resuspended in an ice-cold Type I collagen/5x Collagen neutralization Buffer (4:1 v/v) ratio) with a concentration of 125.000 cells/10 µL. The 5x collagen neutralization buffer is composed of 2.5 g alpha MEM powder (5x) and 2% (w/v) NAHCO3 in 45 mL Aqua dest. and 5 mL of 1M HEPES and set to pH 7.5. 10 µL domes of collagen were plated in 6-well multiwell plates. The domes polymerized for 45 minutes at 37°C. Afterwards basal medium was added. The collagen domes were cultured overnight until transplantation. For orthotopic transplantation, the mice were shaved and anesthetized with 2.5% (v/v) isoflurane. Analgesia was guaranteed by intraperitoneal injection with buprenorphine. The mouse was placed on its back on a heating pad and legs were fixed with leukoplast. Isoflurane was lowered to 1.8% (v/v). A 10 – 15 mm incision was made along the linea alba to open the abdomen. The cecum was placed on a wet sterile gauze. A 3 – 4 mm incision through the cecal serosa was made at the end of the cecum in an area without vessels. The serosa was separated from the submucosal layer and a deep pocket was formed. The pocket was enlarged to the size that the collagen dome could be deeply embedded in it. The collagen dome was placed under the serosa in the pocket and the serosa was tightly closed above the collagen dome securing the collagen dome to be tightly embedded in its pocket. The incision was covered with Seprafilm (Baxter, USA). The cecum was carefully placed back in the abdomen. The peritoneum and abdominal wall were separately closed by a continuous suture. The mice were placed on drinking water supplemented with metamizole (5 mg/mL) for 3 days.

### RT-PCR

RNA was extracted by RNeasy Mini Kit (Qiagen) and was reverse-transcribed to cDNA with oligo(dT)16 primer (Qiagen, QuantiTect Reverse Transcription Kit) according to the manufacturer´s protocol. The cDNA served as template for amplification of target genes, as well as the housekeeping genes by real-time PCR. Intron-spanning primer sets were used throughout all experiments and were designed with Primer 3 Input (https://primer3.ut.ee/). Quantitative RT-PCR was performed with the primer described in the **Supplementary Table 1** using the Qiagen SybrGreen RT PCR Kit (Qiagen) and the LightCycler480 (Roche, Switzerland) according to the manufacturer´s protocol. The LightCycler480 software was used to obtain second derivative crossing point values and relative expression of target genes was calculated by comparative method after normalization to housekeeping gene expression.

### RNAseq

Mouse APTAK tumor tissue (primary tumors n=3, PC n=3) was minced with a rotor-stator-homogenizer (IKA, fisher scientific, USA). RNA was extracted with RNeasy Mini kit (Qiagen). RNAseq was performed by Genewiz, Leipzig, Germany. Hereby cDNA sequencing libraries were prepared using poly(A) mRNA. The libraries were sequenced as 2x150 bp paired end using the Illumina NovaSeq instrument (Illumina, USA).

For bioinformatics analysis of RNAseq data, reads were aligned to the mm39 genome with STAR v2.7.3a (30) using -outSAMtype BAM SortedByCoordinate –outSAMunmapped Within –outSAMattributes Standard –readFilesCommand gunzip as parameters. Counts were obtained using featureCounts v2.0.0 (31) using -p -B -C -Q 10 –primary -T 8 -s 0 as parameters. Differential expression analysis was performed using DESeq2 v1.32.0 (32) using variance stabilization transform normalization.

### Gene Set Ontology Analysis

Gene Set Ontology Analysis (GSEA) was carried out using GSEA v 3.0 (33), using weighted statistic, difference of classes (from pseudo-log_2_+1 tranformed data), gene set permutation and median-division normalization.

### Data Availability

All RNA-Seq data used in this study were deposited at the Gene Expression Omnibus (34) under accession GSE202454.

### Isolation of Tumor Cells and Leucocytes From Mouse and Human Tissue

Mice were killed and primary tumors (n=27), liver metastases (n=15) and PC (n=16) were retrieved in ice-cold PBS. Tumors were minced with scissors and dissociated enzymatically with the tumour dissociation kit mouse, (Miltenyi) and mechanically with the gentleMACS tissue dissociator (Miltenyi, Germany) according to the manufacturer’s instructions. The resulting single cell suspension was filtered through a 100 µm cell strainer (Greiner Bio-One) and erythrocytes were lysed with ACK lysis buffer (Gibco). Cells were counted and either used for organoid culture or FACS staining.

Human samples for FACS analysis were excised during routine pathological examination from patients with tumor surgery in 2021. Primary CRC samples (n=5), liver metastases (n=5) and PC (n=5) were cut in small pieces and enzymatically and mechanically dissociated with the tumor dissociation kit human (Miltenyi) and the gentleMACS tissue dissociator (Miltenyi) according to the manufacturer´s protocol. After dissociation, the resulting single cell suspension was filtered through a 70 µm cell strainer and erythrocytes were lysed with ACK lysis buffer (Gibco).

### Flow Cytometry

Isolated human and mouse single cell suspension were either stimulated for cytokine analysis or directly proceeded with antibody staining. For stimulation, cells were stimulated with Cell Activation cocktail (Biolegend) for 3 hours and Brefeldin A was added for another 2 hours. Cells were first stained with live/dead staining with Zombie NIR Fixable Viability kit (Biolegend) and Fc receptors were blocked with Fc block TruStain FcX™ (anti-mouse CD16/32) antibody (Biolegend). Cells were then incubated with fluorescence-labelled antibodies (**Supplementary Table 1**) for surface staining in FACS buffer (PBS, 2% BSA) for 20 minutes at 4°C. For intracellular staining, cells were fixed in fixation buffer (Foxp3 staining buffer, Invitrogen) for 1 hour and washed two times in permeabilization buffer. Cells were stained with intracellular antibodies (**Supplementary Table 1**) for 20 minutes at 4°C in permeabilization buffer, washed once in permeabilization buffer, and analyzed on a BD LSRFortessa™ flow cytometer (BD Biosciences, USA).

### Immunohistochemistry

Human primary tumour tissue (n=5) and PC tissue (n=50) and mouse tissue from the orthotopic organoid mouse CRC model (primary tumor n=4, liver metastases n=4 and PC n=4) were harvested, fixed in 4% paraformaldehyde for 24 hours and embedded into paraffin.

Briefly, slides were deparaffinized with Rotihistol (Carl Roth), rehydrated in a descending alcohol series, and finally washed with PBS and water. Antigen retrieval was performed by cooking the slides in 20mM Citrate Buffer (pH 6.0) or Tris-EDTA (pH 9.0). To reduce endogenous peroxidase activity the slides were incubated with 3% H_2_O_2_ for 30 minutes. Background staining was reduced by incubating the slides in goat serum (Sigma-Aldrich) followed by incubation with the primary anti-mouse or anti-human antibody at 4°C overnight (**Supplementary Table 1**). Next day slides were washed and primary antibodies were detected using goat anti-rabbit or goat-anti-mouse biotinylated secondary antibody (abcam) according to the manufacturer’s instructions. Streptavidin hrp (abcam) and DAB plus (Zytomed Systems) was added, the slides were washed with H_2_O, counterstained with haematoxylin and mounted with Rotihistokit (Carl Roth). Tumor slides were visualized by Axio Scan.Z1 (Carl Zeiss, Germany) with 20x magnification scan. Positive-stained cells were quantified based on five high-power fields each representing characteristics of the whole tumor slide. Cells were counted by two independent examiners with ImageJ (Wayne Rasband, National Institutes of Health, USA = Rasband, W.S., ImageJ, U. S. National Institutes of Health, Bethesda, Maryland, USA, https://imagej.nih.gov/ij/, 1997-2018.).

### Immunofluorescence

Organoids were stained as previously described (35). APTAK organoids were seeded in 40 µL Cultrex Reduced Growth Factor Basement Membrane Extract, Type 2 (R&D Systems, Bio-Techne) in 8-well Millicell^®^ EZ SLIDES (Merck) and incubated for at least two days in organoid culture medium. Proliferation was analyzed by EdU incorporation (Invitrogen) 6 h prior to fixation. Organoids were fixed in 4% Paraformaldehyde in PBS at room temperature for 20 minutes. Fixative was removed and organoids were washed once in IF buffer (PBS, 0.2% Triton X-100, 0.05% Tween). Then 300 µL of permeabilization solution (PBS, 0.5% Triton X-100) was added for 30 minutes at room temperature. After permebilization, the organoids were blocked in blocking solution (IF buffer, 1% BSA) for 30 minutes at room temperature. EdU visualization *via* Click-iT cocktail was performed prior to primary antibody staining following the Invitro click-iT staining protocol (Invitrogen). Primary antibodies (**Supplementary Table 1**) were incubated overnight in a humidified chamber at 4°C. After overnight incubation, chamberslides were washed three times with IF buffer and the appropriate secondary antibody was added for 1 hour at room temperature. After incubation, secondary antibody solution was removed and organoids were stained for DNA with DAPI (1 µg/mL) in IF buffer for 5 minutes. Chambers were then washed three times with IF buffer, chambers were detached and slides were covered in fluorescence mounting medium (DAKO) and analyzed with a Zeiss LSM880 microscope (Carl Zeiss, Germany).

### RNA *In-Situ* Hybridization

*In-situ* Hybridization (ISH) was conducted using RNAscope^®^ (Advanced Cell Diagnostics by Bio-Techne) for Lgr5. Staining was performed using RNAscope^®^ 2.5 HD Detection Reagent BROWN and RNAscope^®^ FastBrown (DAB) Kit. Supplied Hs-UBC and dapB served as negative and positive control respectively. Experiments were performed according to the supplied manual. Epitope recovery was performed as described above. Nuclear staining was performed with Mayer’s hematoxylin (Sigma-Aldrich) diluted 1:5 in Aqua dest.

instead of haemalum according to Gill I. Tumor slides were visualized by Axio Scan.Z1 (Carl Zeiss, Germany) with 20x magnification scan. Positive-stained cells were quantified based on five HPF with Zen Blue (Carl Zeiss, Germany). Cells were counted by two independent examiners with ImageJ (Wayne Rasband, National Institutes of Health, USA = Rasband, W.S., ImageJ, U. S. National Institutes of Health, Bethesda, Maryland, USA, https://imagej.nih.gov/ij/, 1997-2018.).

### SA-β-Gal Activity Assay in Cryosections

SA-β-gal activity was measured with the senescence β-galactosidase staining kit (Cell Signaling Technology) as recommended by the manufacturer. Briefly, 20 µm serial cryosections from human liver metastases (n=3), human PC (n=3), mouse primary APTAK tumors (n=5), APTAK liver metastases (n=5) and APTAK PC (n=5) were washed with PBS and fixed in fixation solution for 10-15 minutes at room temperature. Then the slides were washed twice in PBS and β-galactosidase staining solution was added. The pH of the β-galactosidase staining solution was adjusted to pH 4.0 (positive control), pH 5.5 (SA-β-gal activity) and pH 7.0 (negative control). Slides were incubated overnight at 37°C in a dry incubator. The stained slides were rinsed in PBS and analyzed using a Zeiss Axio Scan.Z1 (Carl Zeiss, Germany) All images were analyzed using Zen Blue software (Carl Zeiss, Germany).

### FACS-Based SA-β-Gal Measurement

Measurement of SA-β-gal activity by FACS analysis was done as described elsewhere (36). In brief, 1x10^6^ single cells isolated from primary tumors, liver metastases and PC were seeded into one well of a 24-well-plate and incubated with 100 nM bafilomycin A1 (Sigma-Aldrich) in serum-free medium at 37°C. Bafilomycin increases the pH in lysosomes to nearly neutral pH. After 1 hour the substrate 5-dodecanoylaminofluorescein-di-b-galactopyranoside (C_12_-FDG, Fisher Scientific) was added at a final concentration of 50 µM. After 2 hours incubation at 37°C senescent cells have converted the non-fluorescent C_12_-FDG to a fluorescent substrate. Finally, cells were washed in PBS and stained for viability with Zombie NIR (Bioloegend) and T cell markers as described in flow cytometry.

### Statistical Analysis

Normal distributed data were evaluated by standard two-tailed Student’s t-tests. Kruskal-Wallis tests or one-way ANOVA were used for data not showing a normal distribution. Statistics were evaluated with GraphPad Prism 9.2.0. GraphPad Prism was used to calculate mean and standard deviation (SD).

## Results

### Human PC Cancer Cells Show Characteristics of Senescence, SASP and Stemness

**Figure 1:**
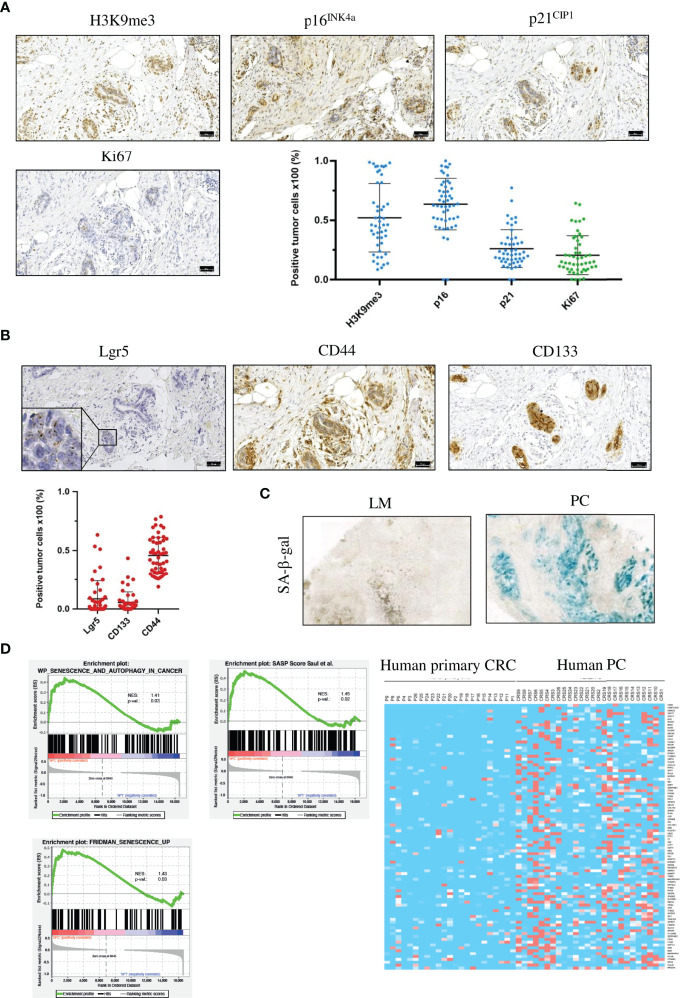
Human PC cancer cells are senescent, have a SASP and show signs of a stem cell-like phenotype. **(A)** Representative images of IHC staining for the senescence-associated markers H3K9me3, p16^INK4a^ and p21^CIP1^ and the proliferation marker Ki67 and quantification of the different markers from one patient. Scale bar: 50 µm, error bars represent the mean ± SD. **(B)** Representative images of IHC staining for the stem cell markers CD44 and CD133 and RNA *in situ* hybridization for Lgr5 from the same patient as in **(A)** Inset represents 125x magnification. Scale bars: 50 µm, error bars represent the mean ± SD. **(C)** Representative images of SA-β-gal in a liver metastasis (left) and PC (right), n = 3 per group. **(D)** Gene set enrichment analysis profiles of senescence and autophagy genes, SASP genes and senescence-associated genes from primary tumors (n = 23) and PC samples (n = 26) from the TCGA PanCancer collection. Heat map of SASP genes from the same patient collective as in the gene set enrichment analysis.

Senescence is a highly heterogeneous state that lacks specific markers (37). We thus first analyzed proliferation and senescence-associated markers in FFPE sections from 50 patients with PC by immunohistochemistry. Senescence is characterized by stable cell-cycle arrest, regulated by the cyclin-dependent kinase (CDK) inhibitors p16^INK4a^ (CDKN2A) and p21^CIP1^ (CDKN1A). High p16^INK4a^ and/or p21^CIP1^ levels, together with a low proliferation index, constitute indicators of senescence. Human PC patient specimens showed a low rate of proliferation marker Ki67 (20% ± 16%, **Figure 1A** and **Supplementary Figure 1A**). Senescence is maintained by epigenetic alterations, notably by trimethylation of histone 3 lysine 9 (H3K9me3). The transcriptionally repressive senescence-associated mark H3K9me3 was detectable in 52% (± 29%) of the tumor cells in PC sections. 63% ( ± 21%) of cancer cells expressed the CDK2/4 inhibitor p16^INK4a^. 26% ( ± 16%) of all tumor cells in PC samples expressed the cell cycle regulator p21^CIP1^ (**Figure 1A** and **Supplementary Figure 1A**). These results indicate that PC is characterized by tumor cells that are predominantly senescent, and that senescence is mainly associated with p16^INK4a^.

Senescent cells can acquire stem cell-like features and resume proliferation. These “previously” senescent cells show pronounced tumor-initiating potential (13). As PC is the most lethal form of CRC, we hypothesized that PC displays stem cell-like characteristics. To test this hypothesis, we stained PC tumor specimen against three different stem cell markers. First, performed staining against the stem cell marker CD44,.a transmembrane glycoprotein that is overexpressed in several cancers (38). In PC sections, 46% (± 15%) of cancer cells were CD44-positive (**Figure 1B** and **Supplementary Figure 1B**), indicating that nearly half of all PC tumor cells expressed this stem cell marker. Next, we analyzed CD133, which is also a transmembrane glycoprotein that is overexpressed in several cancers (39). In human PC sections, 5% (± 9%) of tumor cells were CD133-positive (**Figure 1B** and **Supplementary Figure 1B**). As third stem cell marker, we used the leucine-rich repeat-containing G-protein coupled receptor 5 (Lgr5). Lgr5 is a target of Wnt and a well-established marker of stem cells in various tissues (40). We detected Lgr5-positive tumor cells in 9% (± 19%) of all PC cancer cells (**Figure 1B** and **Supplementary Figure 1B**). To analyze the correlation between senescence-associated markers and stem cell markers, we applied Spearman correlation analysis. H3K9me3 showed a strong correlation with Lgr5 (r = 0.60, p = 0.000007), moderate correlation (r = 0.39, p = 0.007) with CD133, and poor correlation (r = 0.29, p = 0.046) with CD44. P16^INK4a^ showed moderate correlation with Lgr5 (r = 0.38, p = 0.009) and CD44 (r = 0.3, p = 0.037) and no correlation (r = -0.15, p = 0.311) with CD133. p21^CIP1^ showed moderate correlation (r = 0.33, p = 0.021) with CD44 (**Supplementary Figure 2**). From the correlation data, we conclude that senescent cancer cells in the peritoneum show features of stemness. To confirm that PC cancer cells, but not liver metastases are senescent, even though originating from the same primary tumor, we measured the activity of the senescence-associated β-galactosidase (SA-β-gal) at pH 6.5. Liver metastases showed no or only faint SA-β−gal activity compared to PC cancer cells, indicating that upregulated expression of senescence-associated markers e.g., p16^INK4a^ is due to senescence induction in the peritoneal cavity (**Figure 1C**). The composition of SASP greatly varies dependent on the cell type and on the inducer of the senescence (41, 42). To investigate SASP in PC and to compare the expression of SASP genes with CRC samples, we performed bioinformatic analysis with publicly available RNA-seq data of 23 primary CRC and 26 PC patients using the TCGA PanCancer collection. Gene Set Enrichment Analysis (GSEA) showed that senescence-associated genes and SASP genes are upregulated in PC data sets compared with primary tumor samples, and that PC samples have a distinct SASP (**Figure 1D**). Several SASP genes were preferentially upregulated only in human PC samples, e.g. VEGF, IFN-γ receptor or CXCL12 compared with primary tumors (**Figure 1D**), showing that senescent PC with stem cell-like features express a unique SASP.

### Peritoneal Metastases Show the Same Senescent Phenotype Both for Human und Murine Tumor Tissue

**Figure 2:**
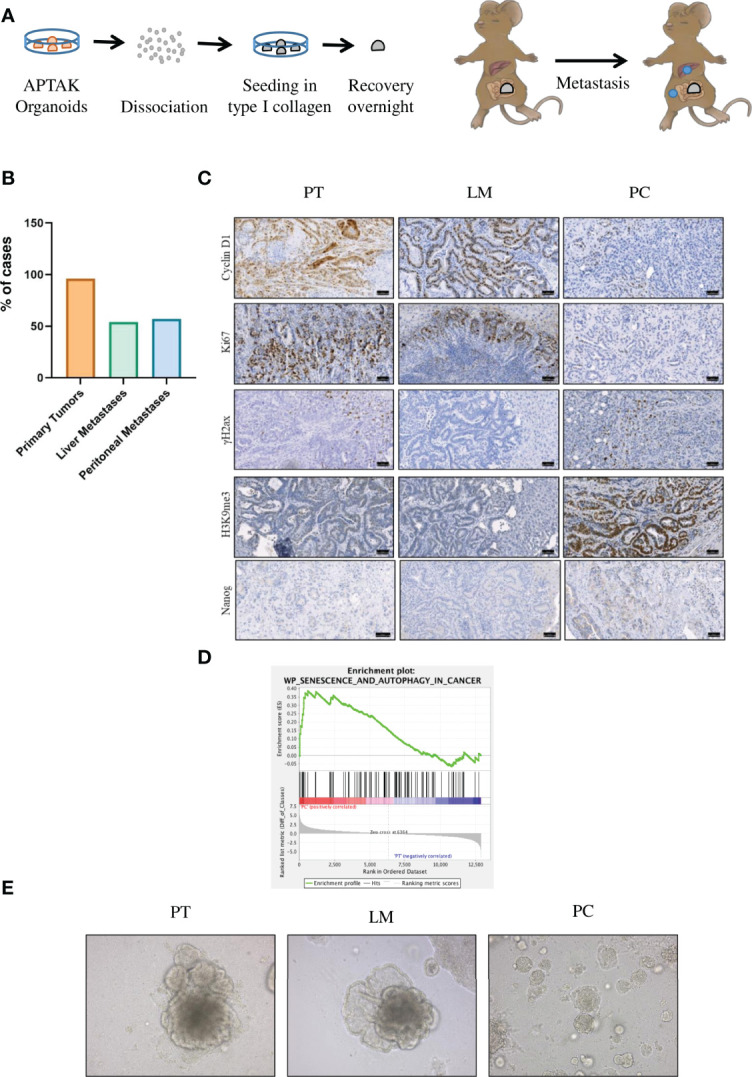
The orthotopic transplantation of APTAK organoids leads to both liver metastases and PC but only cancer cells in the peritoneal cavity show induction of senescence. **(A)** Schematic protocol of the orthotopic transplantation of APTAK organoids under the serosa of the cecum. **(B)** Quantification of primary tumors (98%, n = 27), liver metastases (54%, n = 15) and PC (57%, n = 16) about 42 days after orthotopic transplantation of APTAK organoids. **(C)** Representative images of IHC staining for the proliferation markers Cyclin D1 and Ki67, the DDR marker γ-H2AX, the senescence marker H3K9me3 and the stem cell marker Nanog in primary tumors (PT), liver metastases (LM) and PC. Scale bar: 50 µm **(D)** Gene set enrichment analysis (GSEA) of senescence and autophagy markers in PC versus primary tumor samples (n = 3 per group). **(E)** Representative images of tumor organoids cultured as 3D culture at d5 derived from tumors (primary tumor (PT), liver metastasis (LM) and peritoneal carcinomatosis (PC)) of the same mouse in the orthothopic organoid transplantation model. Magnification 20X.

Due to genetic alterations, APTAK organoids are cancer cells with a high metastatic potential To obtain a mouse model of PC, we transplanted APTAK organoids under the serosa of the cecum of C57Bl/6J mice (25) (**Figure 2A**). Transplantation of 125,000 APTAK cells leads to liver and peritoneal metastasis after about 42 days. This long latency *in vivo* gives the opportunity to study metastases of liver and peritoneal cavity in depth. **Figure 2A** shows the protocol of orthotopic transplantation. After about 42 days, we sacrificed the animals and isolated primary tumors, liver metastases and peritoneal metastases. 98% of animals had primary tumors (n=27), 54% liver metastases (n=15) and 57% peritoneal metastases (n=16), or both (**Figure 2B**).

**Figure 3:**
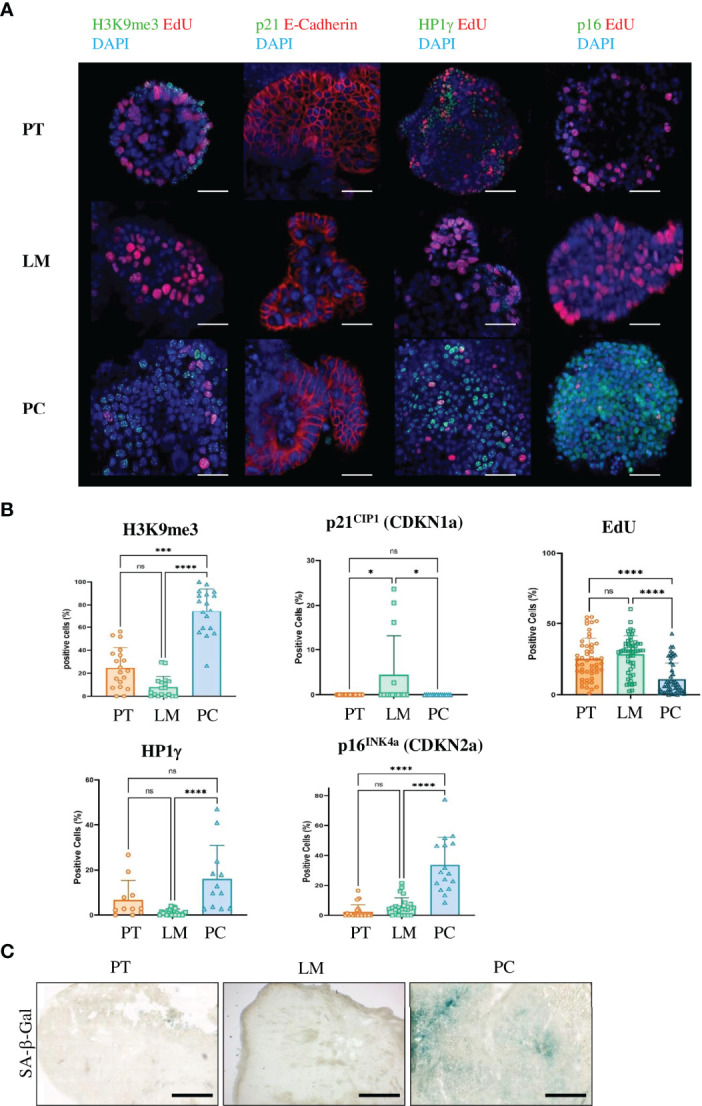
Only cancer cells from metastases in the peritoneal cavity of the APTAK mouse model show a senescent phenotype. **(A)** Representative immunofluorescence (IF) images of the senescence-associated markers H3K9me3, p21^CIP1^, HP1γ and p16^INK4a^ (all green) from organoids derived from primary tumors (PT), liver metastases (LM) and PC from the orthotopic organoid transplantation CRC mouse model. The proliferation marker EdU was stained in red and nuclei were stained in blue. p21 was combined with the cell membrane-bound E-Cadherin (red) and not with EdU. Scale bars represent 50 µm. **(B)** Quantification of IF markers per group derived from primary tumors (n = 5 mice), liver metastases (n = 3 mice) and PC (n = 3 mice) from the orthotopic organoid transplantation CRC mouse model. 3-5 HPF per mouse and marker with at least 1000 APTAK cancer cells were analyzed. Error bars represent the mean ± SD. Kruskal-Wallis test **(B)** ****p < 0.0001, ***p < 0.0002, *p<0.05, n.s. = not significant. **(C)** Representative images of SA-β-gal from primary tumors (PT), liver metastasis (LM) and peritoneal carcinomatosis (PC) from a mouse of the orthotopic organoid mouse CRC model. n = 5 mice per group. Scale bars represent 1000 µm.

To confirm that the mouse model resembles human peritoneal carcinomatosis, we analyzed the induction of senescence. We first performed immunohistochemistry for the proliferation markers Cyclin D1 and Ki67 in FFPE sections from primary tumors, liver metastases and PC. Cancer cells in primary tumors and liver metastases were highly proliferative as shown by positive staining with Ki67 and Cyclin D1 antibodies in contrast to PC sections (**Figure 2C** and **Supplementary Figures 3A, B**). Senescent cells show nuclear alterations as a result from activation of DNA damage response (DDR) pathways, leading to phosphorylation of histone H2AX. Sections from PC showed γ-H2AX-positive staining, however liver metastases and primary tumor sections were also positive for this marker, although to a lesser extent (**Figure 2C** and **Supplementary Figure 4**). To confirm that the peritoneal cavity is a metastatic niche where senescence is induced, we went on to stain against the senescence-associated marker H3K9me3. This analysis revealed that the repressive epigenetic mark H3K9me3 was exclusively expressed in PC sections, whereas primary tumors and liver metastases showed no staining (**Figure 2C** and **Supplementary Figure 5**). As human PC sections showed marked upregulation of stem cell markers, we analyzed the expression of another stem cell marker Nanog. PC sections showed upregulated Nanog expression, while primary tumors and liver metastases showed no expression (**Figure 2C** and **Supplementary Figure 6**). To confirm the immunochemistry results, we performed GSEA analysis. Consistent with findings in human PC genes, senescence and autophagy genes were markedly upregulated in PC samples from our mouse model (**Figure 2D**). Since we could not exclude that in TME but not cancer cells were senescent in PC, we isolated cancer cells from primary tumors, liver metastases and peritoneal metastases and cultured them as 3D tumor organoids. In culture, organoids derived from PC showed reduced proliferation compared with organoids from liver metastases or primary tumors (**Figure 2E**), indicating that the metastatic niche transforms tumor cells. After one passage, we performed immunofluorescence staining (**Figure 3A**). PC tumor cells showed significantly lower proliferation (as seen by the EdU staining) compared with primary tumors (p<0.0001) and liver metastases (p<0.0001). We also observed significantly higher expression of the senescence-associated marker H3K9me3 compared with primary tumors (p = 0.0002) and liver metastases (p<0.0001). p16^INK4a^ (CDKN2a) was significantly more expressed in PC organoids than in primary tumors (p<0.0001) and liver metastases (p<0.0001). Only the heterochromatin protein 1 gamma (HP1γ) was not significantly upregulated in PC compared with primary tumors (p = 0.1435) (**Figure 3B**). In most cases, senescence is induced by p16^INK4a^ or by the transcription factor p53 and its downstream target p21. However, p21 can induce senescence independently of p53, although to a much lesser extent. As APTAK tumor cells harbor a mutation in the p53 protein, we expected very little to no expression of p21 in the nuclei of senescent cells. To avoid having three different stainings (DAPI in blue, EdU in red and p21 in green) in one nucleus, we swapped EdU staining with the cell membrane-bound E-Cadherin. The senescence marker p21^CIP1^ was significantly higher in liver metastases compared with primary tumors (p = 0.0101) and PC (p = 0.0206) but not significantly upregulated in PC compared with primary tumors (p = 0.9999). (**Figures 3A, B**). By evaluating enzyme activity in cryosections of PC tumors, liver metastases and primary tumors, we were able to demonstrate that primary tumors and liver metastases showed only faint X-Gal staining compared with PC (**Figure 3C**). Our results show that in the case of senescence, orthotopic transplantation of APTAK organoids resembles the clinical situation in human PC. In conclusion, the peritoneal cavity represents a metastatic niche that induces senescence, whereas the same tumor cells show no signs of senescence induction within the metastatic environment of the liver.

### Peritoneal Metastases After Orthotopic Transplantation Show Stem Cell and SASP-Like Phenotypes

**Figure 4:**
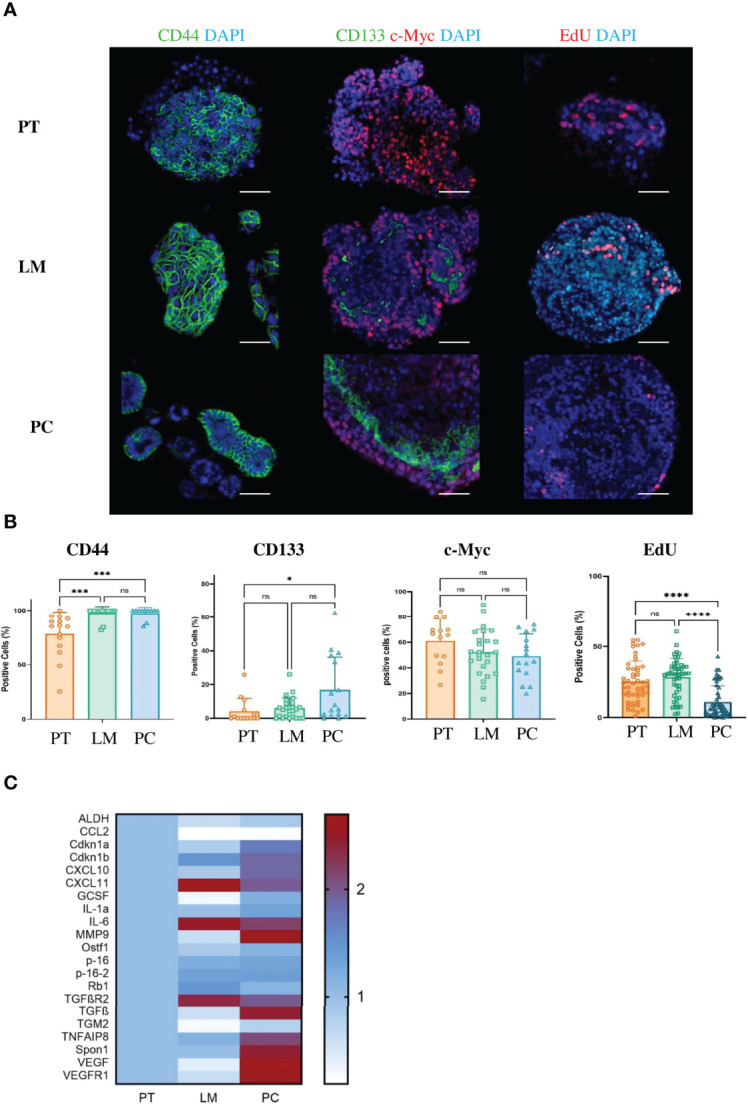
Cancer cells from metastases in the peritoneal cavity of the APTAK mouse model show enhanced stem cell-like phenotype and a SASP. **(A)** Representative immunofluorescence (IF) images of the stem cell markers CD44, CD133 (green) and c-Myc (red) of tumor organoids derived from primary tumors (PT), liver metastases (LM) and PC from the orthotopic organoid transplantation CRC mouse model. The proliferation marker EdU stained in red and nuclei in blue. Scale bars represent 50 µm. **(B)** Quantification of IF markers from **(A)**. 5 HPF with at least 1000 cells from primary tumors (n = 5), liver metastases (n = 3) and PC (n = 3) were counted. Error bars represent the mean ± SD. Kruskal-Wallis test **(B)** ****p < 0.0001, ***p < 0.0002, *p < 0.05, n.s. = not significant. **(C)** Heatmap of characteristic SASP genes from tumor organoids derived from primary tumors (PT), liver metastases (LM) and PC (n = 3 mice per group) from the orthotopic organoid transplantation CRC mouse model. Tumors were isolated, dissociated and cultured for one passage as 3D organoids. Then mRNA was isolated and qPCR was performed.

Regarding the analyzed patient samples, more than half of tumor cells acquired stem cell-like features with markers such as CD44, CD133, and Lgr5 (**Figure 1B**). To validate that our CRC metastasis model also resembles the clinical situation in terms of stem cell-like characteristics, we stained primary tumor cells, liver metastases and PC with antibodies against the stem cell markers CD44, CD133, and c-Myc (**Figure 4A**). As expected, PC tumor cells showed enhanced expression of CD44 (p = 0.0007) and CD133 (p = 0.0224) together with significantly reduced proliferation rate (p<0.0001) as compared to primary tumor cells, whereas the upregulation ofc-Myc was not significant (p = 0.2826) (**Figure 4B**). Thus, our mouse model truly recapitulates the situation of CRC patients with disseminated disease. SASP varies between cell types and according to the inducer of senescence (42).. To analyze the SASP phenotype in murine PC, we performed qPCRs for several genes that are typically expressed in senescent epithelial cells. Some of these genes such as Vascular Endothelial Growth Factor (VEGF), Vascular Endothelial Growth Factor Receptor 1 (VEGFR1), Spondin 1 (Spon1), Transforming Growth Factor beta (TGFβ) or Metalloproteinase 9 (MMP9) were exclusively upregulated in cancer cells from the peritoneal cavity (**Figure 4C**). These results show that although cancer cells acquire a stem cell-like phenotype in PC, they still express SASP.

### Peritoneal Carcinomatosis Harbors a Tumor Microenvironment With Senescence-like T Cells

**Figure 5:**
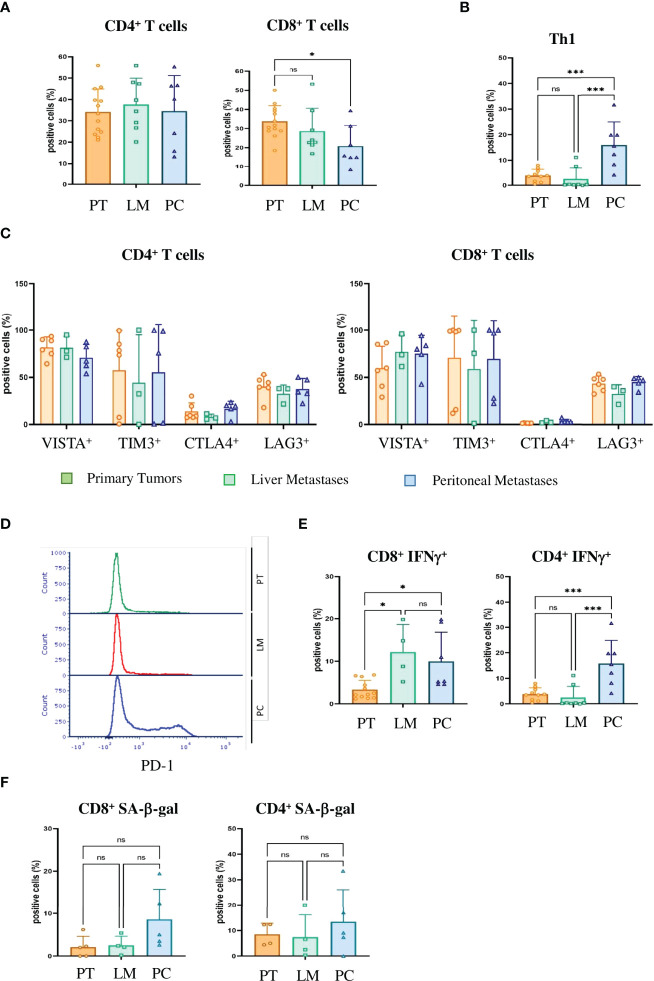
Tumor-infiltrating T cells in PC express checkpoint inhibitor molecules, IFN-γ and markers of senescence and thus are immunosenescent **(A)** Flow cytometric analysis of the tumor infiltrating lymphocytes of primary tumors (PT), liver metastases (LM) and PC of the orthotopic organoid transplantation CRC mouse model for CD4^+^ and CD8^+^ T cells. Dotplots show the percentage of CD4^+^ and CD8^+^ T cells gated on CD3^+^ T cells (n = 13 for PT, n = 8 for LM, n = 7 for PC). **(B)** The number of IFN-γ secreting T helper 1 (Th1) cells evaluated by FACS in the tumor microenvironment of primary tumors (PT), liver metastases (LM) and PC of the orthotopic organoid transplantation CRC mouse model. Dotplots show the percentage of IFN-γ secreting CD4^+^ T cells gated on live CD45^+^ CD3^+^ T cells (n = 13 for PT, n = 8 for LM, n = 7 for PC). **(C)** Flow cytometric analysis of CD4^+^ (left panel) and CD8^+^ (right panel) T cells for checkpoint molecules VISTA, Tim3, CTLA4 and Lag3 in tumor infiltrating lymphocytes of primary tumors (PT), liver metastases (LM) and PC of the orthotopic organoid transplantation CRC mouse model. Dotplots show the percentage of the CD4^+^ and CD8^+^ T cells expressing the respective immune checkpoint molecule (n = 8 for PT, n = 3 for LM, n = 4 for PC). **(D)** Flow cytometric analysis of the expression of PD1 on tumor-infiltrating CD8^+^ T cells from primary tumors (PT), liver metastases (LM) and PC of the orthotopic organoid transplantation CRC mouse model. **(E)** Flow cytometric analysis from tumor-infiltrating CD8^+^ and CD4^+^ T cells for IFN-γ secretion of primary tumors (PT), liver metastases (LM) and PC of the orthotopic organoid transplantation CRC mouse model. Dotplots show the percentage of IFN-γ positive CD4^+^ and CD8^+^ T cells (n = 12 for PT, n = 4 for LM, n = 7 for PC). **(F)** Flow cytometric analysis of SA-β-gal positive tumor-infiltrating CD8^+^ and CD4^+^ T cells in the tumor microenvironment of primary tumors (PT), liver metastases (LM) and PC of the orthotopic organoid transplantation CRC mouse model. Dotplots show the percentage of SA-β-gal CD4^+^ and CD8^+^ T cells (n = 5 for PT, n = 4 for LM, n = 5 for PC). Error bars represent the mean ± SD. One-way ANOVA **(A–C, E, F)** ***p < 0.0002, *p<0.05, n.s. = not significant. In all analysis Zombie NIR staining was used to discriminate dead from live cells and only live Zombie-negative cells were quantified.

Previously, we could show that T-cell infiltration of human peritoneal metastases is significantly lower than in corresponding primary colorectal cancer samples (10). To verify whether our PC mouse model clinically mimics tumor-infiltrating lymphocytes (TILs), we analyzed PC-, liver metastasis- and primary tumor-derived TILs by FACS. Primary tumor, liver metastasis and PC samples showed similar infiltration of CD45-positive leukocytes and CD3-positive T-cells. There were minor differences in the amount of CD4-positive T-helper cells, but a significant reduction in the amount of CD8-positive T cells in PC compared with primary tumors (p = 0.0287) (**Figure 5A**). In a previous study, immunohistochemistry of surgical PC specimens from patients showed significant upregulation of interferon-gamma (IFN-γ)-positive CD4^+^ T-helper cells (Th1 cells) compared with primary tumors (10). FACS staining with IFN-γ and CD4 similarly revealed significant upregulation of Th1 TILs in our mouse PC samples compared with primary tumors (p = 0.0007) and liver metastases (p = 0.0003) (**Figure 5B**).

**Figure 6:**
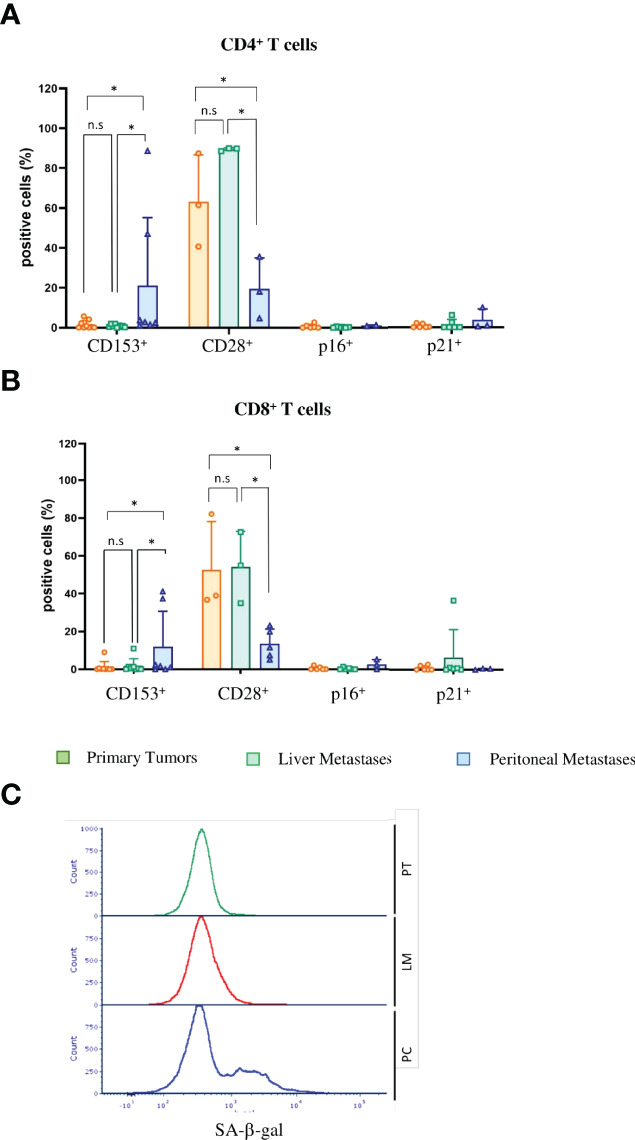
Tumor-infiltrating lymphocytes in PC show signs of immunosenescence in mice and men **(A)** Flow cytometric analysis of immunosenescence associated markers on tumor-infiltrating CD4^+^ T cells of primary tumors (PT), liver metastases (LM) and PC of the orthotopic organoid transplantation CRC mouse model. Dotplots show the percentage of CD4^+^ T cells expressing CD153 (n = 10 for PT, n = 7 for LM, n = 8 for PC), CD28 (n = 3 for PT, n = 3 for LM, n = 3 for PC), p16 and p21 (n = 10 for PT, n = 7 for LM, n = 8 for PC). **(B)** Flow cytometric analysis of immunosenescence associated markers on tumor-infiltrating CD8^+^ T cells of primary tumors (PT), liver metastases (LM) and PC of the orthotopic organoid transplantation CRC mouse model. Dotplots show the percentage of CD8^+^ T cells expressing CD153 (n = 10 for PT, n = 7 for LM, n = 8 for PC), CD28 (n= 3 for PT, n=3 for LM, n=3 for PC), p16 and p21 (n= 10 for PT, n = 7 for LM, n = 8 for PC). **(C)** Flow cytometric analysis of SA-β-gal in tumor-infiltrating lymphocytes from human patients with primary CRC (PT), liver metastasis and PC. The histogram shows the results of 5 patients each. Error bars represent the mean ± SD. One-way ANOVA **(A, B)** *p < 0.05, n.s. = not significant.

SASP factors can induce senescence in neighboring cells, referred as paracrine senescence. This “bystander” senescence is in part mediated by TGFβ and VEGF (43). Since we obtained marked upregulation of TGFβ and VEGF only in PC cells and not primary tumor or liver metastasis cells (**Figure 4C**), we wondered whether senescent PC cells could induce senescence in TILs. Senescent and exhausted T-cells share several overlapping characteristics. Both express checkpoint inhibitor molecules, including programmed cell death protein 1 (PD-1), cytotoxic T-lymphocyte-associated protein 4 (CTLA-4), T-cell immunoglobulin and mucin-domain containing-3 (Tim3) and V-domain Ig suppressor of T-cell activation (VISTA) (44). In our mouse model, CD4-positive T-cells as well as CD8-positive T-cells expressed high levels of VISTA, Tim3, Lag3 and PD-1, independent of the compartment (**Figures 5C, D**). However, unlike exhausted T-cells that downregulate metabolic function, senescent T-cells still produce high amounts of inflammatory cytokines such as Interleukin 2 (IL-2), Interleukin 6 (IL-6), Tumor Necrosis Factor (TNF) and IFN-γ. CD4^+^ and CD8^+^ PC T-cells expressed significantly higher levels of IFN-γ compared with primary tumor T-cells (p = 0.039 and p = 0.0235) (**Figure 5E**). Together with increases in inflammatory cytokines, the upregulation of checkpoint inhibitor molecules indicates that not only tumor cells but also infiltrating T-cells become senescent in PC. We next performed SA-β-gal analysis to verify that T-cells are not merely exhausted but actually senescent. We saw no significant upregulation of SA-β-gal activity in either CD4^+^ T cells (p = 0.999) or CD8^+^ T cells (p = 0.0746) (**Figure 5F**). In humans, T-cells downregulate or lose the co-stimulatory molecules CD27 and CD28 independently of the type of senescence (45). In contrast, senescent murine T-cells often maintain expression of CD28 and CD27 (44) and express the tumor necrosis factor ligand superfamily member 8 molecule (CD153). In our CRC metastasis mouse model, significantly increased numbers of tumor-infiltrating CD4-positive T-cells isolated from PC metastasis were CD153-positive as compared to those from originating from liver metastasis (p = 0.031) or primary tumors (p = 0.0454)(**Figure 6A**). In CD8^+^ T-cells from PC samples, there was also a significant upregulation of CD153^+^ compared with primary tumors (p = 0.03) and liver metastasis (p = 0.038) (**Figure 6B**). We also found significantly reduced CD28 expression in CD4^+^ T-cells from PC as compared with primary tumors (p = 0.0325) and liver metastasis (p = 0.0219). PC-derived CD8^+^ T-cells showed significant downregulation of CD28 compared with primary tumors (p = 0,0607) and liver metastasis (p = 0.0255) (**Figure 6B**). Finally, we isolated infiltrating leukocytes from human primary CRC (n = 5), liver metastasis (n = 5) and PC (n = 5) samples and measured SA-β-gal activity by FACS analysis. Only PC-derived tumor-infiltrating cells showed features of senescence whereas primary tumor and liver metastasis showed no SA-β-gal activity (**Figure 6C**).

Altogether, these results indicate that senescent PC cells induce senescence in tumor-infiltrating leukocytes in both patients and PC mouse model.

## Discussion

Cellular senescence was first described in fibroblasts, where sustained cell duplication shortens telomere length and induces permanent cell cycle arrest, known as replicative senescence. Cells that are no longer able to divide cannot become highly proliferative cancer cells, showing the potential of senescent cells as a barrier against malignant transformation. Therefore senescence, a state of permanent cell cycle arrest, has been considered an intrinsic mechanism of cancer suppression (8, 46–49). Mice that lack the pro-apoptotic protein Bak have no phenotype and do not develop tumors as compared with wildtype animals (50). In contrast, inactivation of the tumor suppressor gene Cdkn2a (encoding p16^Ink4a^) in mice leads to early formation of tumors that is not observed in wildtype littermates (51), respectively. These studies could show that senescence induction is, at least in these mouse models, a more important form of tumor control than apoptosis. Here, we show that in human patients, the peritoneal niche induces senescence in disseminated CRC cells, which instead of arresting further tumor progression leads to contradictory tumor growth. Irreversible growth arrest was long considered a hallmark of senescence that distinguishes senescence from quiescence, a transient form of growth arrest (52). However, some studies demonstrated that in therapy-induced senescence, senescent cells can escape from cell cycle arrest and give rise to even more aggressive cancer. Achutan et al. showed that treatment of multiple breast cancer cell lines with doxorubicin leads to escape from senescence by a small population of cancer cells. These senescence escapers exhibit stem cell characteristics and express increased levels of the stem cell marker CD133 (53). CD133 upregulates the WNT signaling pathway *via* PI3K/AKT pathway, increasing stem cell properties (39). A recent study by the group of Clemens Schmitt showed that the chemotherapeutic Adriamycin (Doxorubicin) induced senescence in mouse lymphoma cells and that these senescent lymphoma cells expressed stem cell markers such as CD133 or CD44. CD44 binds hyaluronate, the main component of the extracellular matrix, linking CD44 expression to cellular adhesion and communication. Cells that escaped senescence and acquired stem cell phenotypes were more aggressive *in vitro* and after implantation *in vivo* (13). Adriamycin-treated colorectal cancer cell lines become senescent and upregulate expression of CD133 and CD44. We could show that in our patient cohort, a substantial amount (46% ± 15%) of PC cancer cells expressed the stem cell marker CD44 and 5% (± 9%) the stem cell marker CD133. Achutan et al. found a such pronounced stem cell phenotype only after *in vitro* treatment with doxorubicin.

To study PC cancer cells in more detail and to compare PC with liver metastasis and primary tumor samples we aimed to introduce a mouse model that clinically mimics human PC. To date, *in vivo* animal studies of CRC rapidly show high tumor burden and mice have had to be sacrificed before metastasis occurred (21–23). To overcome this problem, we adopted an orthotopic transplantation model (25) and adapted this model with APTAK tumor organoids for metastasis in the peritoneal cavity. APTAK organoids recapitulate the transcriptome-based consensus molecular subtype (CMS) 4 of the CMS classification of colorectal cancer (54). We found marked upregulation of senescent cancer cells in both mouse and human PC samples. One hallmark of senescence, independent of the inducer of senescence, is that senescent cells secrete multiple factors including pro-inflammatory cytokines, chemokines, growth factors and proteases (55). Some of these factors are known to induce “bystander” senescence in neighboring cells (56). In mouse PC samples, we found the same upregulation of senescence-associated markers accompanied by downregulation of proliferation as in human samples. Furthermore, we detected the same upregulation of senescent cancer cells with a stem cell phenotype in human and in mouse PC samples. Previously, we could show that infiltration of CD4^+^ T-cells in human PC specimens is significantly reduced compared with primary tumor samples (10). The group Ohira group also showed that infiltration of CD8^+^ T cells in peritoneal metastasis is also reduced compared with liver and lung metastasis (57). In our mouse model, the degree of CD8^+^ T-cell infiltration was significantly reduced compared with primary tumor and liver metastasis samples, showing that our new mouse model truly recapitulates the clinical setting of human peritoneal carcinomatosis.

In our study, we observed that although about 25% of PC cancer cells from our PC mouse model had a stem cell phenotype. These cells still uniquely expressed SASP factors. *Via* qPCR we measured the expression of SASP factors that can induce bystander senescence in neighboring cells, e.g. VEGF and TGFβ (20). Induction of paracrine senescence by SASP factors is not limited to cancer cells. Many solid tumors progress rapidly, although large numbers of T-cells can still infiltrate. One reason for this discrepancy is T-cell senescence, and another might be T-cell exhaustion (58). In both states, checkpoint inhibitor molecules such as PD-1 or Tim3 are upregulated but unlike senescent T cells, exhausted T cells do not produce pro-inflammatory cytokines such as TNF or IFN-γ (58). In the mouse model, PC TILs showed expression of checkpoint inhibitor molecules and production of TNF and IFN-γ, indicating that T-cells are dysfunctional and show signs of senescence. A hallmark of senescent T-Cells in human samples is the dramatic reduction of the co-stimulatory molecules CD27 and CD28 (45). However, in mice this reduction is not always observed. Instead, upregulation of CD153 is a marker of T cell senescence in mice (44). Unexpectedly, in the peritoneal metastasis TME, we observed both. I.e, (i) a significant downregulation of CD28 and (ii) upregulation of CD153 compared with liver metastases and primary tumors. As our mouse model indicated that senescent cancer cells in the peritoneal cavity spread senescence not only to other non-senescent cancer cells *via* SASP, but induced a senescence-like phenotype in tumor-infiltrating lymphocytes, we analyzed tumor-infiltrating leukocytes in human samples. Only leukocytes isolated from human PC samples showed upregulation of SA-β-gal activity, indicating that in human PC, senescent cancer cells induce senescence in TILs. Senescent tumor cells that induce senescence in immune TME cells *via* secretion of SASP factors seem represent a more widespread phenomenon in aggressive tumors that are resistant to immune checkpoint blockade. In the most aggressive brain cancer, the Glioblastoma multiforme (GBM), patients displayed not only senescent tumor cells but also senescent immune cells in the tumor microenvironment and systemically in peripheral blood (27–29). In this context, a recent study by Puca et al. could show that treatment of senescent glioblastoma U87-MG cells and senescent T-cells derived from peripheral blood of GBM patients with the senolytic drug longevity-associated variant (LAV) of the bactericidal/permeability-increasing fold-containing family B member 4 (LAV-BPIFB4) reduced the senescent phenotype in both U87-MG cells and patient-derived PBMCs (29). In our PC model, treatment of mice with a senolytic drug may represent a successful approach to reduce senescence in PC cells and in immune TME cells and prevent the development of cancer cells with stem cell characteristics. Depleting senescent cancer stem cells could thus restore the beneficial effects of chemotherapeutics. Reducing senescence in immune cells could at least decrease resistance to immunotherapies with checkpoint inhibitors. One important limitation to successfully translate therapies such immunotherapies or senotherapeutics to the clinic is the lack of animal models that fully recapitulate the human situation. We think that our PC mouse model clinically mimics human PC. This gives us the opportunity to study the effects and results of e.g., the novel one-two punch cancer therapy approach. In this novel cancer therapy, the first punch, e.g. chemotherapy, kills cancer cells. Nevertheless, this treatment induces senescence in cancer cells. The first punch is then followed by the removal of senescent cells by senotherapeutics, the second punch. This new therapeutic approach is still experimental and there are many open questions to be answered (59).

The inducer of cancer cell senescence in the peritoneal cavity is unknown. Given that liver metastasis and PC APTAK organoids express the same oncogene, Kras^G12D^, but that liver cancer cells display no senescence hallmarks, we can probably rule out oncogene-induced senescence (OIS) in the orthotopic mouse model. Unlike in PC patients, in the PC model, animals were not treated with chemotherapeutics, indicating that neither therapy-induced senescence (TIS) nor OIS are the sole inducer. Given that in PC, the level of IFN-γ is upregulated and the peritoneal cavity harbors peritoneal macrophages that secrete high levels of TNF, we suspect cytokine-induced senescence as the inducer (8, 9, 60).

About one third of all CRC patients acquire peritoneal metastasis after the initial diagnosis (61). The prognosis for CRC patients with PC is poor, and most patients do not benefit from systemic chemotherapy (3). To date, the only successful treatment option is cytoreductive surgery, as a clinical phase III study showed that combination with hyperthermic intraperitoneal chemotherapy was not superior to surgery alone (6). Several cytotoxic drugs show increased activity with higher temperature and thus better penetrate in mild hyperthermia (4). This was the rationale for HIPEC therapy. Most chemotherapeutics rely on proliferating cells, as most chemotherapeutic drugs target cells at different phases of the cell cycle. In our study, we could show that most human PC cancer cells became senescent with strongly reduced proliferation rates. This could account for resistance of PC against chemotherapy. In addition, although senescent cells usually do not divide anymore, they are still viable and resistant to apoptosis. Local treatment of senescent PC cells with HIPEC might force even more cells into a stem cell-like phenotype, rendering PC metastasis even more aggressive and resistant to any other anti-cancer treatment except surgery.

Altogether, in our study, we could show that the peritoneal cavity is a unique metastatic niche that induces senescence in cancer cells in both patients and mice. Up to 40% of these senescent cancer cells acquired a stem cell-like phenotype. This can have serious therapeutic consequences, as cancer stem cells are resistant to most forms of chemotherapy (13). In addition, senescent cells secrete a multitude of SASP factors that can induce senescence in neighboring TME cells. In our mouse model, we could show that infiltrating T-cells upregulate checkpoint inhibitor molecules as well as senescence-associated molecules, indicating that T-cells were not exhausted but senescent. In human PC samples, we could show that tumor-infiltrating immune cells show signs of senescence as measured by upregulated SA-β-gal activity, whereas immune cells isolated from the primary tumors and liver metastases show no senescence induction. This might represent another important therapeutic problem, as senescent immune cells are unaffected by checkpoint inhibitor therapy.

## Data Availability Statement

The datasets presented in this study can be found in online repositories. The names of the repository/repositories and accession number(s) can be found below: NCBI GEO, accession no: GSE202454.

## Ethics Statement

The studies involving human participants were reviewed and approved by Ethics committee Regensburg No. 14-101-0014 Ethics committee Freiburg No. 21-1162. The patients/participants provided their written informed consent to participate in this study. The animal study was reviewed and approved by Regierungspräsidium Freiburg G19/041.

## Author Contributions

HB and RK conceived and conducted the project. HB wrote the manuscript, PC and CB performed bioinformatics, HB and DP performed experiments, CL helped in establishment of the mouse model, CB, EB, and PH helped in clinical evaluation of PC patients. All authors reviewed the manuscript. All authors contributed to the article and approved the submitted version.

## Funding

Deutsche Forschungsgemeinschaft (FOR2438, KE2164/1-1) for RK supported the work.

## Conflict of Interest

The authors declare that the research was conducted in the absence of any commercial or financial relationships that could be construed as a potential conflict of interest.

## Publisher’s Note

All claims expressed in this article are solely those of the authors and do not necessarily represent those of their affiliated organizations, or those of the publisher, the editors and the reviewers. Any product that may be evaluated in this article, or claim that may be made by its manufacturer, is not guaranteed or endorsed by the publisher.

## Supplementary Material

Supplementary Figure 1
**(A)** Representative images of immunohistochemical (IHC) staining for the senescence-associated markers H3K9me3, p16^INK4a^ and p21^CIP^ and the proliferation marker Ki67 from 5 different PC patients. Scale bar: 50 µm **(B)** Representative images of immunohistochemical (IHC) staining for the stem cell markers CD44, CD133 and Lgr5 from 5 different PC patients. Scale bar: 50 µm, 20 µm (Lgr5).

Supplementary Figure 2 Pearson coefficients correlation for the senescence-associated markers H3K9me3, p16^INK4a^, p21^CIP1^ and the stem cell marker Lgr5, CD133 and CD44 from 50 different PC patients.

Supplementary Figure 3
**(A)** Representative images of immunohistochemical (IHC) staining for the proliferation marker Ki67 from primary tumors (PT), liver metastasis (LM) and peritoneal carcinomatosis (PC) from 4 different mice of the orthotopic organoid mouse CRC model. Scale bar: 50 µm. **(B)** Representative images of immunohistochemical (IHC) staining for the proliferation marker Cyclin D1 from primary tumors (PT), liver metastasis (LM) and peritoneal carcinomatosis (PC) from 4 different mice of the orthotopic organoid mouse CRC model. Scale bar: 50 µm.

Supplementary Figure 4 Representative images of immunohistochemical (IHC) staining for senescence-associated marker γH2ax from primary tumors (PT), liver metastasis (LM) and peritoneal carcinomatosis (PC) from 4 different mice of the orthotopic organoid mouse CRC model. Scale bar: 50 µm.

Supplementary Figure 5 Representative images of immunohistochemical (IHC) staining for senescence-associated marker H3K9me3 from primary tumors (PT), liver metastasis (LM) and peritoneal carcinomatosis (PC) from 4 different mice of the orthotopic organoid mouse CRC model. Scale bar: 50 µm.

Supplementary Figure 6 Representative images of immunohistochemical (IHC) staining for stem cell marker Nanog from primary tumors (PT), liver metastasis (LM) and peritoneal carcinomatosis (PC) from 3 different mice of the orthotopic organoid mouse CRC model. Scale bar: 50 µm.
