# Supplementary figures and images for: Senescent Tumor Cells in the Peritoneal Carcinomatosis Drive Immunosenescence in the Tumor Microenvironment
Source: Front Immunol. 2022 Jun 30;13:908449. doi: 10.3389/fimmu.2022.908449 (PMC9279937; doi:10.3389/fimmu.2022.908449)

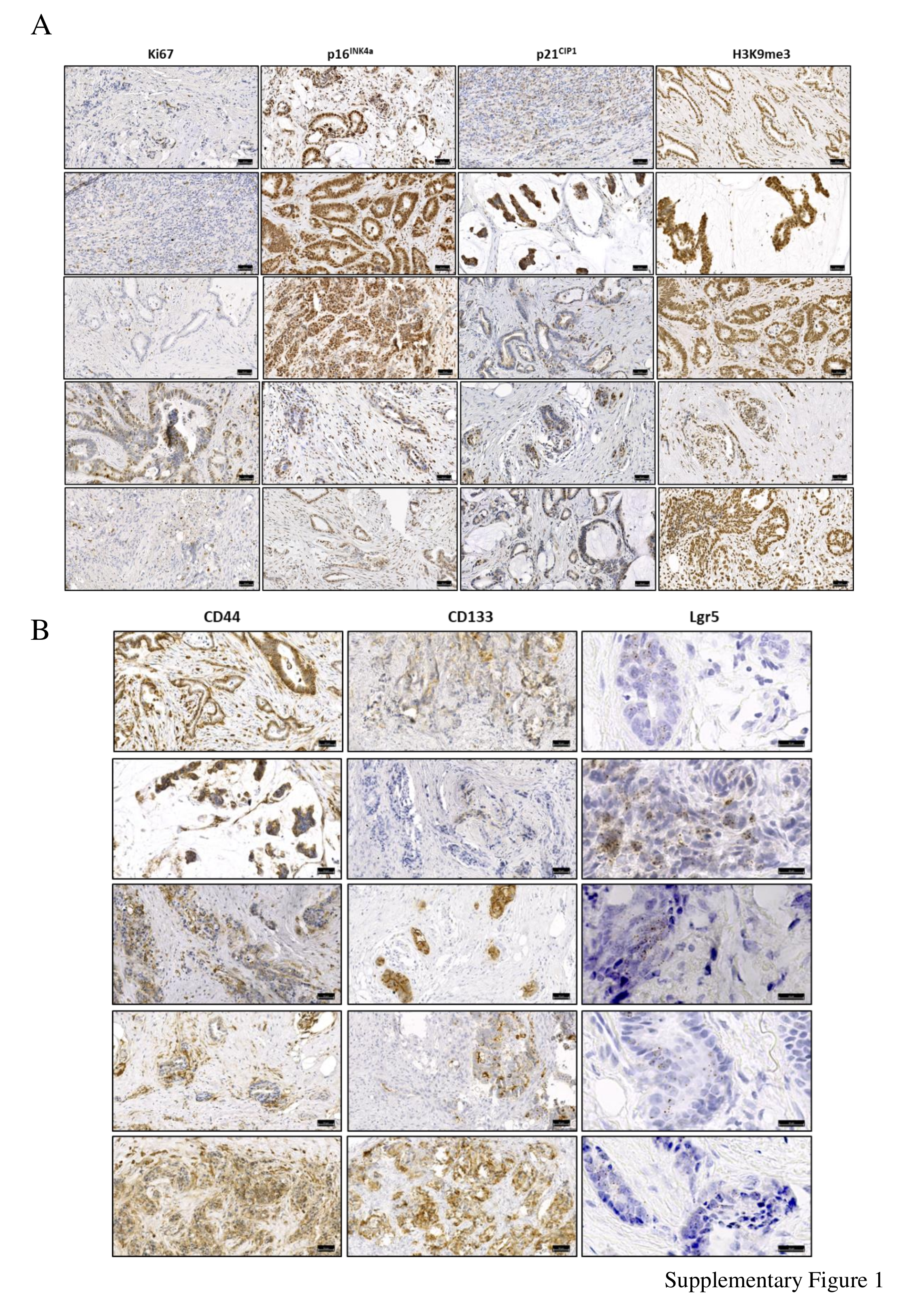

Supplement: Supplementary Figure 1 — (A) Representative images of immunohistochemical (IHC) staining for the senescence-associated markers H3K9me3, p16INK4a and p21CIP and the proliferation marker Ki67 from 5 different PC patients. Scale bar: 50 µm (B) Representative images of immunohistochemical (IHC) staining for the stem cell markers CD44, CD133 and Lgr5 from 5 different PC patients. Scale bar: 50 µm, 20 µm (Lgr5). [file Image_1.tif]

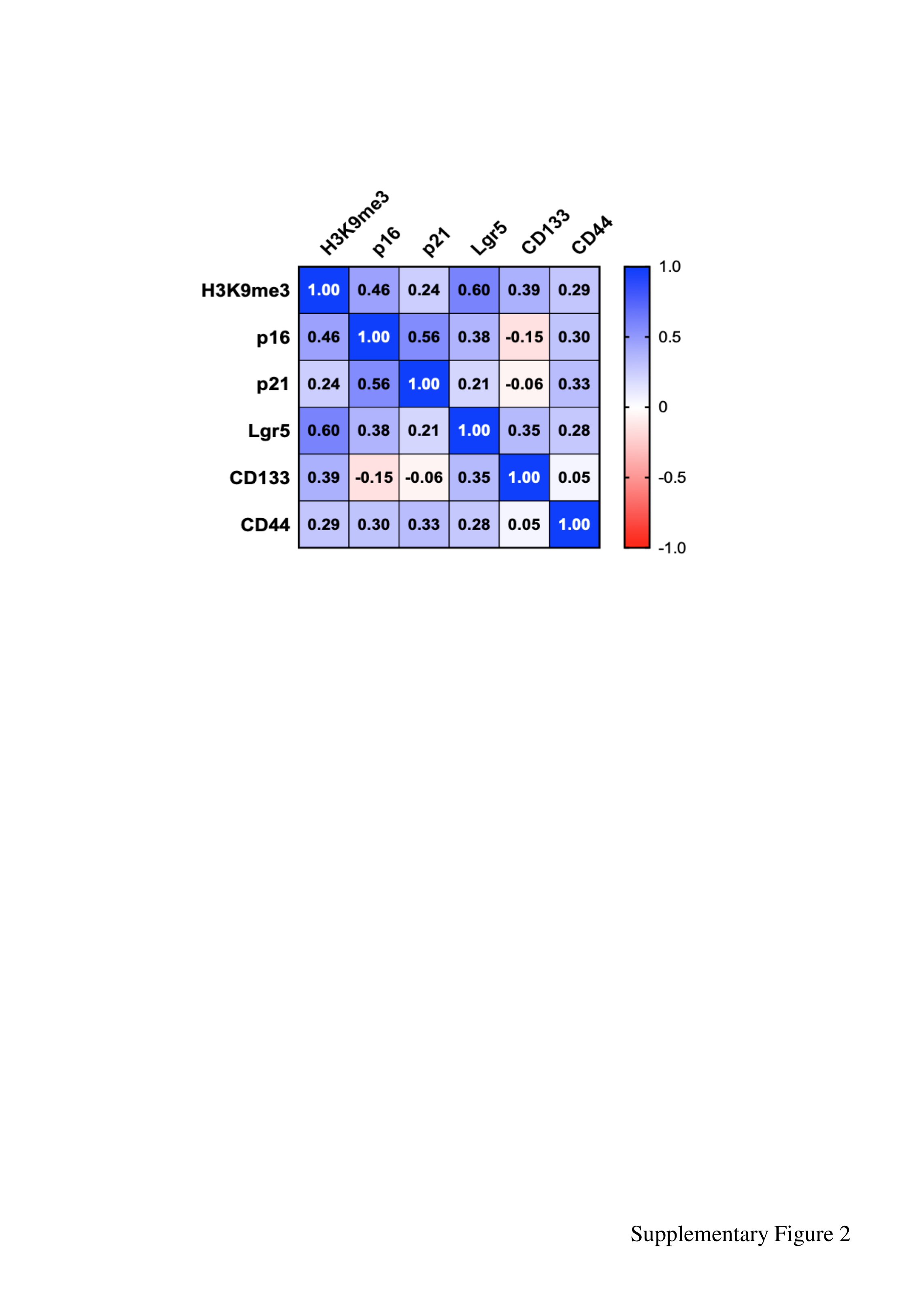

Supplement: Supplementary Figure 2 — Pearson coefficients correlation for the senescence-associated markers H3K9me3, p16INK4a, p21CIP1 and the stem cell marker Lgr5, CD133 and CD44 from 50 different PC patients. [file Image_2.tif]

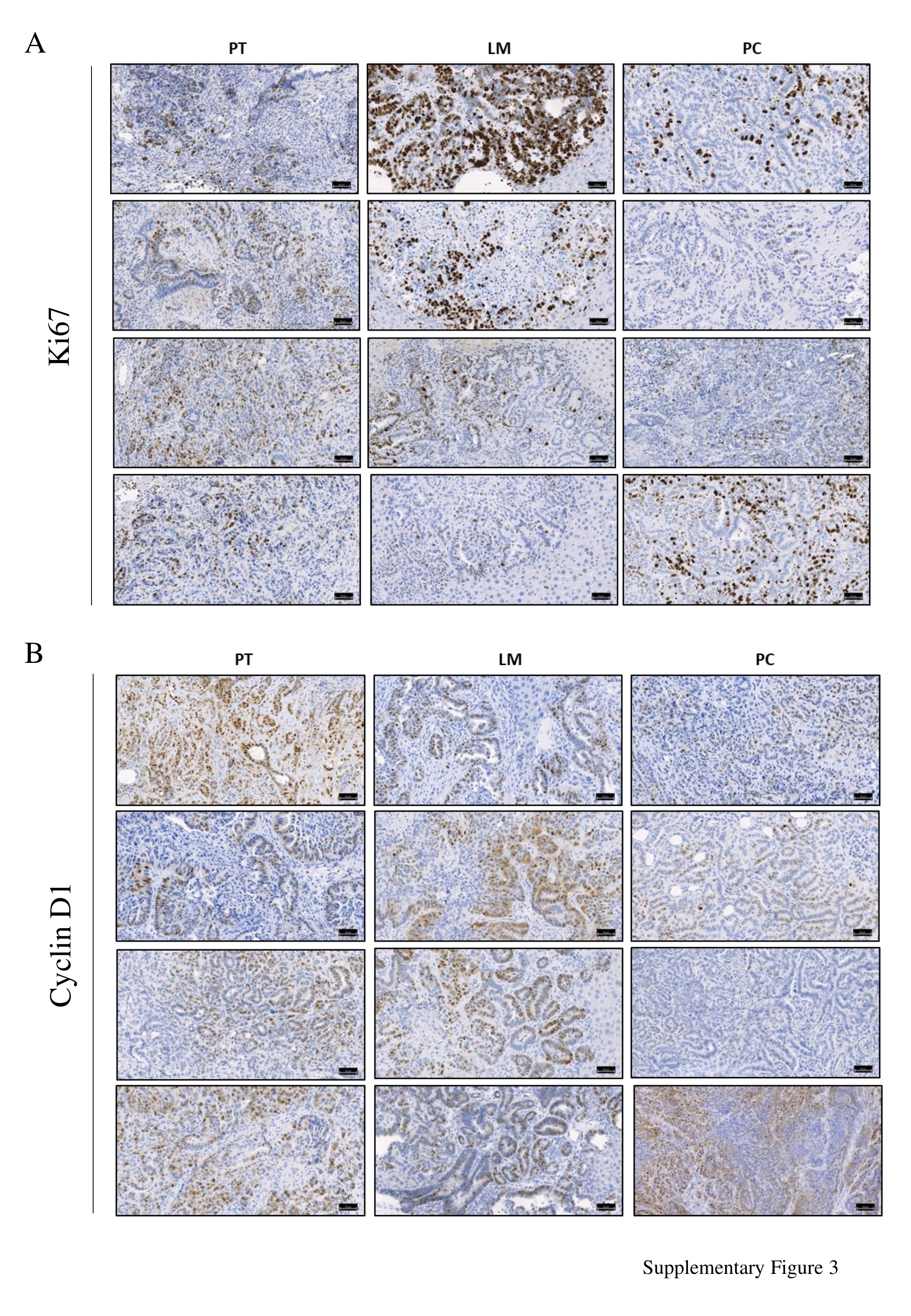

Supplement: Supplementary Figure 3 — (A) Representative images of immunohistochemical (IHC) staining for the proliferation marker Ki67 from primary tumors (PT), liver metastasis (LM) and peritoneal carcinomatosis (PC) from 4 different mice of the orthotopic organoid mouse CRC model. Scale bar: 50 µm. (B) Representative images of immunohistochemical (IHC) staining for the proliferation marker Cyclin D1 from primary tumors (PT), liver metastasis (LM) and peritoneal carcinomatosis (PC) from 4 different mice of the orthotopic organoid mouse CRC model. Scale bar: 50 µm. [file Image_3.tif]

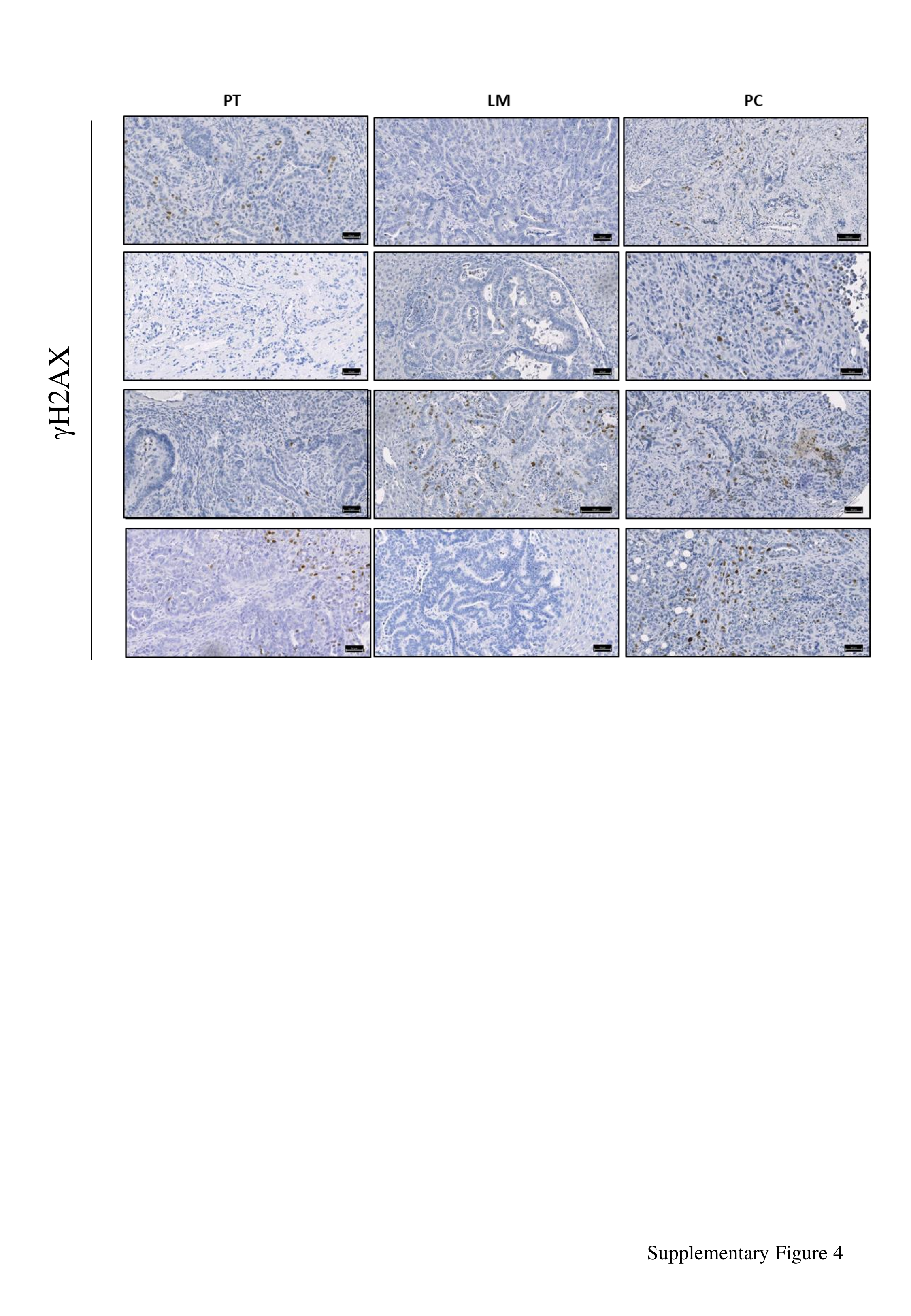

Supplement: Supplementary Figure 4 — Representative images of immunohistochemical (IHC) staining for senescence-associated marker γH2ax from primary tumors (PT), liver metastasis (LM) and peritoneal carcinomatosis (PC) from 4 different mice of the orthotopic organoid mouse CRC model. Scale bar: 50 µm. [file Image_4.tif]

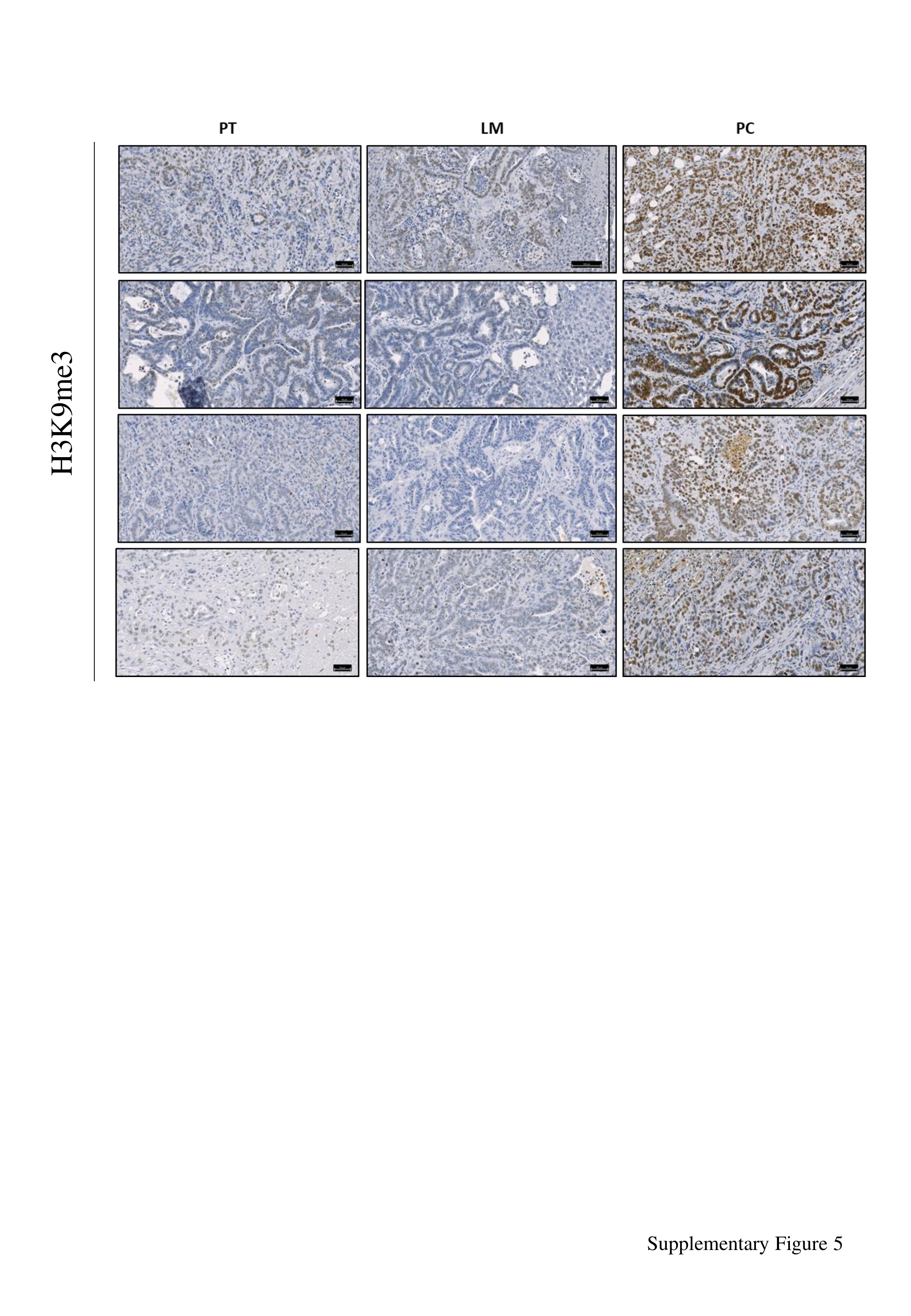

Supplement: Supplementary Figure 5 — Representative images of immunohistochemical (IHC) staining for senescence-associated marker H3K9me3 from primary tumors (PT), liver metastasis (LM) and peritoneal carcinomatosis (PC) from 4 different mice of the orthotopic organoid mouse CRC model. Scale bar: 50 µm. [file Image_5.tif]

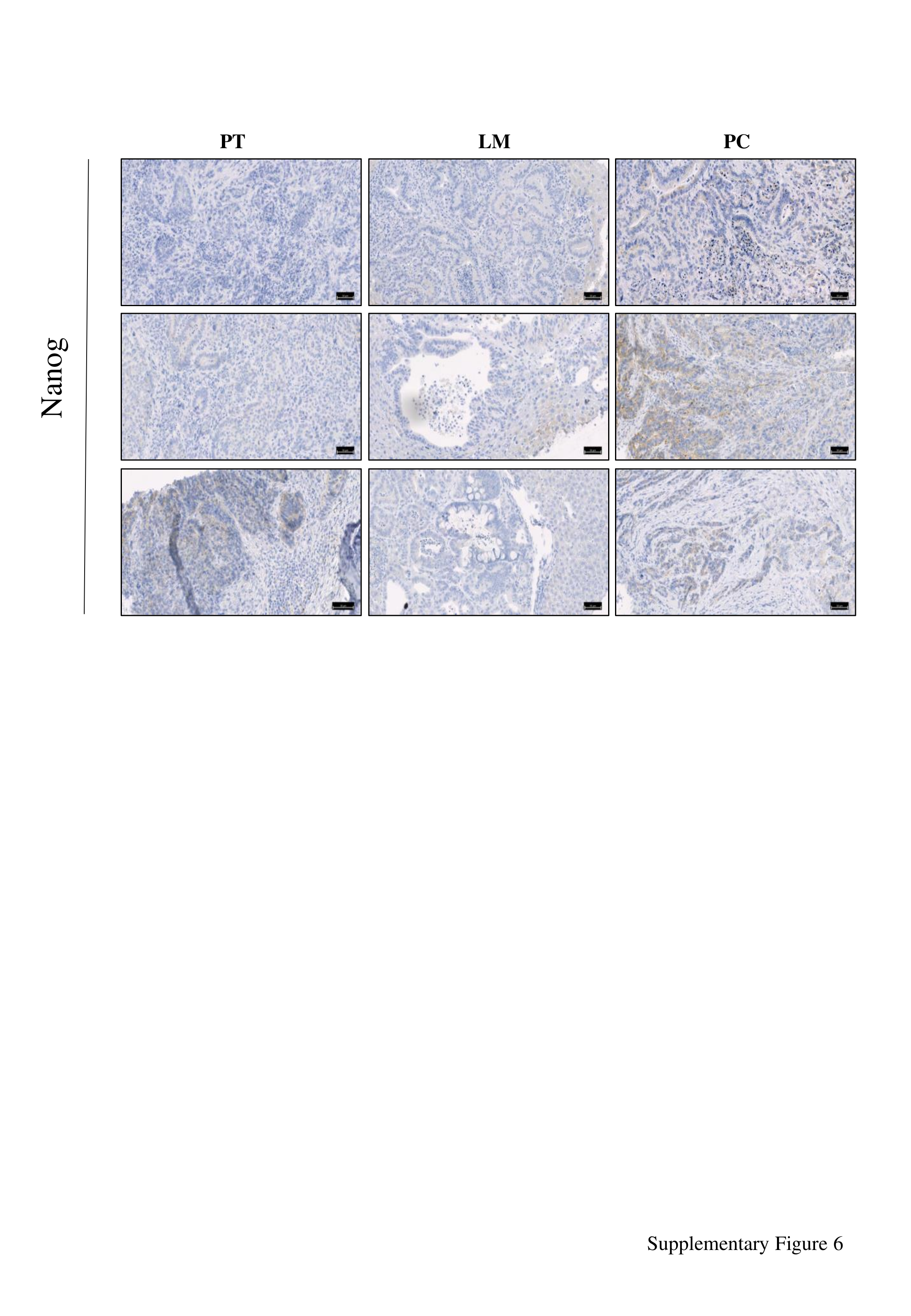

Supplement: Supplementary Figure 6 — Representative images of immunohistochemical (IHC) staining for stem cell marker Nanog from primary tumors (PT), liver metastasis (LM) and peritoneal carcinomatosis (PC) from 3 different mice of the orthotopic organoid mouse CRC model. Scale bar: 50 µm. [file Image_6.tif]
