# Supplementary material for: Senescent Tumor Cells in the Peritoneal Carcinomatosis Drive Immunosenescence in the Tumor Microenvironment
Source: Front Immunol. 2022 Jun 30;13:908449. doi: 10.3389/fimmu.2022.908449 (PMC9279937; doi:10.3389/fimmu.2022.908449)
Supplement: Supplementary file 7 [file Table_1.docx]

Table S1

List of antibodies and primers

| Antibodies IF and FACS | Source | Dilution | Identifier |
| --- | --- | --- | --- |
| Rabbit-anti-E-Cadherin | Cell Signaling | 1:200 | Cat# 3195 |
| Rabbit-anti-c-Myc | Cell Signaling | 1:1600 | Cat# 13987 |
| Rabbit-anti-CDKN2a | BioRad | 1:200 | Cat# AHP_1488 |
| Mouse-anti-p21 | Santa Cruz | 1:800 | Cat# sc-53870 |
| Rabbit-anti-H3K9me3 | GeneTex | 1:100 | Cat# GTX54103 |
| Rabbit-anti-HP1gamma | Abcam | 1:1000 | Cat# ab217999 |
| Rat-anti-CD133 | Invitrogen | 1:200 | Cat# 53-1331-80 |
| Rat-anti-CD44 | BD Pharmingen | 1:50 | Cat# 550538 |
| Donkey-anti-rabbit AF488 | ThermoFisher Scientific | 1:500 | Cat# A-32790 |
| Donkey-anti-mouse AF488 | ThermoFisher Scientific | 1:500 | Cat# A-32766 |
| Donkey-anti-rat AF488 | ThermoFisher Scientific | 1:500 | Cat# A-48269 |
| Donkey-anti-rabbit AF568 | ThermoFisher Scientific | 1:500 | Cat# A-11011 |
| Anti-mouse CD45 AF700 | Biolegend | 1:500 | Cat# 103127 |
| Anti-mouse CD3 BV421 | Biolegend | 1:500 | Cat# 100227 |
| Anti-mouse CD4 PerCP-Cy5.5 | Biolegend | 1:500 | Cat# 100433 |
| Anti-mouse CD8b PE-Dazzle594 | Biolegend | 1:500 | Cat# 126621 |
| Anti-mouse CD153 APC | Biolegend | 1:500 | Cat# 106409 |
| Anti-mouse PD-1 BV510 | Biolegend | 1:500 | Cat# 135241 |
| Anti-mouse IFN-g BV785 | Biolegend | 1:500 | Cat# 505837 |
| Anti-mouse TNF BV650 | Biolegend | 1:500 | Cat# 506333 |
| Anti-mouse IL-17A AF488 | Biolegend | 1:500 | Cat# 517005 |
| Anti-mouse Foxp3 AF488 | Biolegend | 1:500 | Cat# 320011 |
| Anti-mouse Tim3 PE-Cy7 | Biolegend | 1:500 | Cat# 134009 |
| Anti-mouse Lag3 BV785 | Biolegend | 1:500 | Cat# 125219 |
| Anti-mouse VISTA PE | Biolegend | 1:500 | Cat# 150203 |
| Zombie NIR | Biolegend | 1:500 | Cat# 423105 |
| Anti-mouse CTLA-4 (CD152) APC | Miltenyi | 1:500 | Cat# 130-102-518 |
| Anti-human TCRgd APC | Miltenyi | 1:500 | Cat# 130-113-508 |
| Anti-human CD45 AF700 | Biolegend | 1:500 | Cat# 368514 |
| Anti-human CD19 | Biolegend | 1:500 | Cat# 302242 |
| Anti-human CD56 BV605 | Biolegend | 1:500 | Cat# 318334 |
| Anti-human CD27 BV711 | Biolegend | 1:500 | Cat# 356430 |
| Anti-human CD4 PerCP | Biolegend | 1:500 | Cat# 317428 |
| Anti-human CD4 PE-Cy7 | Biolegend | 1:500 | Cat# 317414 |
| Anti-human Lag3 BV510 | Biolegend | 1:500 | Cat# 369318 |
| Anti-human TIGIT BV605 | Biolegend | 1:500 | Cat# 372712 |
| Anti-human CD39 BV711 | Biolegend | 1:500 | Cat# 328228 |
| Anti-human PD-1 PerCP | Biolegend | 1:500 | Cat# 621614 |
| Anti-human Tim3 BV650 | Biolegend | 1:500 | Cat# 345028 |
| Anti-human CTLA-4 BV421 | Biolegend | 1:500 | Cat# 369606 |
| Anti-human CD8 PE-Dazzle594 | Biolegend | 1:500 | Cat# 344744 |
| Anti-human CD3 BV785 | Biolegend | 1:500 | Cat# 344842 |

| Antibodies IHC | Source | Dilution | Identifier |
| --- | --- | --- | --- |
| Rabbit Anti-CD44 | abcam | 1:10000 | Cat# ab51037 |
| Rabbit Anti-CD133 | abcam | 1:5000 | Cat# ab222782 |
| Rabbit Anti-Cyclin D1 | Cell Signaling | 1:50 | Cat# 55506 |
| Rabbit Anti-γ-H2A.X | Cell Signaling | 1:300 | Cat# 9718 |
| Rabbit Anti-Histone H3 (tri methyl K9) | abcam | 1:2500 | Cat# ab8898 |
| Rabbit Anti-Ki67 | Cell Signaling | 1:300 | Cat# 9129 |
| Rabbit Anti-Nanog | Cell Signaling | 1:250 | Cat# 8892 |
| Rabbit Anti-PCNA | Cell Signaling | 1:4000 | Cat# 13110 |
| Rabbit Anti-p16^INK4a^ (CDKN2A) | LSBio | 1:2000 | Cat# LS‑B1347 |
| Rabbit Anti-p21 | abcam | 1:1000 | Cat# ab232512 |

| Software, algorithms and databases | Source | Identifier |
| --- | --- | --- |
| ImageJ | Rasband, W.S., ImageJ, U. S. National Institutes of Health, Bethesda, Maryland, USA, https://imagej.nih.gov/ij/index.html | NA |
| Cell_counter ImageJ plugin | Kurt De Vos | NA |
| DESeq2 v1.32.0 | Love et al. | NA |
| featureCounts | Liao et al. | NA |
| FlowJo | BD Biosciences | NA |
| GSEA 4.1.0 | Broad Institute | NA |
| GraphPad Prism 9.2.0 | GraphPad Software | NA |
| LightCycler^®^ 480 SW 1.5.1 | Roche | NA |
| Primer3 | https://primer3.ut.ee | NA |
| STAR v2.7.3a | Dobin et al. | NA |
| The Cancer Genome Atlas Pan-Cancer analysis project | The Cancer Genome Atlas Research Network et al. | NA |
| Zen 2 (blue edition) | Zeiss | NA |

List of primers

| Primer name | Primer sequence |
| --- | --- |
| Aldh18a left | CTGGCTACCTTAGAGCCTGAAC |
| Aldh18a right | GGATCTCTTCCCGCTGGT |
| Ccl2 left | CATCCACGTGTTGGCTCA |
| Ccl2 right | GATCATCTTGCTGGTGAATGAGT |
| Cdkn1a_p21 left | TCCACAGCGATATCCAGACA |
| Cdkn1a_p21 right | GGACATCACCAGGATTGGAC |
| Cdkn1b_p27 right | TCTGTTCTGTTGGCCCTTTT |
| Cdkn1b_p27 left | GTTAGCGGAGCAGTGTCCA |
| Cdkn2a_p16 left | GGGTTTTCTTGGTGAAGTTCG |
| Cdkn2a_p16 right | TTGCCCATCATCATCACCT |
| p16complete left | GGGTTTTCTTGGTGAAGTTCG |
| p16complete right | TTGCCCATCATCATCACCT |
| Cxcl11 left | GCGGCTGCTGAGATGAAC |
| Cxcl11 right | CGCCCCTGTTTGAACATAAG |
| Cxcl10 left | CTTGCCTGAGCCTAACCAA |
| Cxcl10 right | TGTCAGTTACGAAATCCTGCAT |
| Actb right | ACCAGAGGCATACAGGGACA |
| Actb left | CTAAGGCCAACCGTGAAAAG |
| Hprt right | CCTGGTTCATCATCGCTAATC |
| Hprt left | TCCTCCTCAGACCGCTTTT |
| Gapdh left | GGGTTCCTATAAATACGGACTGC |
| Gapdh right | CCATTTTGTCTACGGGACGA |
| Rps18 left | AAGTGTCTTCTGCTCACTCAAGG |
| Rps18 right | GATCACTCGCTCCACCTCAT |
| Tgm2 left | ACCTGCTGGCTGAGAGAGAT |
| Tgm2 right | GGTTTTGCTTGGGTTCTCC |
| IL1a left | TTGGTTAAATGACCTGCAACA |
| IL1a right | GAGCGCTCACGAACAGTTG |
| IL6 right | CCAGGTAGCTATGGTACTCCAGAA |
| IL6 left | GCTACCAAACTGGATATAATCAGGA |
| Mip1_ccl3 right | GTGGAATCTTCCGGCTGTAG |
| Mip1_ccl3 left | TGCCCTTGCTGTTCTTCTCT |
| MMP9 left | AGACGACATAGACGGCATCC |
| MMP9 right | TCGGCTGTGGTTCAGTTGT |
| Rb1 left | TTCCAACAGAAACTGGCAGA |
| Rb1 right | AGCCAGGAGTCTGGTGTCC |
| Ostf1 left | AGTTGCTACTGGCAAAAGGTG |
| Ostf1 right | TTCTTCAGGAGCGATGCAC |
| Spon1 left | CATGATGCCTGAGTGCCATA |
| Spon1 right | ACGCTACAGTCGCTCCACTC |
| Vegfa left | TTAAACGAACGTACTTGCAGATG |
| Vegfa right | AGAGGTCTGGTTCCCGAAA |
| Tnfaip8 left | ACTCCGGGGAACAGCATT |
| Tnfaip8 right | TGGCCAGTCCCTAGAGAGG |
| Tgfb2 left | AGGAGGTTTATAAAATCGACATGC |
| Tgfb2 right | TAGAAAGTGGGCGGGATG |
| VEGFR1 for | CCTCACCGTGCAAGGAAC |
| VEGFR2 rev | GTGATGTACACGATGCCATGC |
| TGFBR2 for | GTCTGTGGATGACCTGGCTAAC |
| TGFBR2 rev | GACATCGGTCTGCTTGAAGGAC |
| GCSF for | TCCAGGAGAAGCTGGTGAGTGA |
| GCSF rev | CGCTATGGAGTTGGCTCAAGCA |
