# Supplementary material for: Senescent Tumor Cells in the Peritoneal Carcinomatosis Drive Immunosenescence in the Tumor Microenvironment
Source: Front Immunol. 2022 Jun 30;13:908449. doi: 10.3389/fimmu.2022.908449 (PMC9279937; doi:10.3389/fimmu.2022.908449)
Supplement: Supplementary file 8 [file Table_2.docx]

Table S2

Clinicopathological characteristics of PC patients

|  | Number | Percentage |
| --- | --- | --- |
| Sex |  |  |
| Female | 19 | 38 |
| Male | 31 | 62 |
| Age |  |  |
| ≤40 | 9 | 18 |
| 41-60 | 25 | 50 |
| 61-80 | 16 | 32 |
| Clinical stage* |  |  |
| Stage I | 3 | 7,4 |
| Stage II | 15 | 36,6 |
| Stage III | 13 | 31,7 |
| Stage IV | 10 | 24,3 |
| Total | 41 | 100 |
| HIPEC |  |  |
| HIPEC with Mitomycin C and Doxorubicin | 7 | 14 |
| HIPEC with 5-FU | 1 | 2 |
| HIPEC with Mitomycin C and Cisplatin | 1 | 2 |
| HIPEC with Palititaxel and Carboplatin | 1 | 2 |
| HIPEC with FOLFOX and Cetuximab | 1 | 2 |
| HIPEC with FOLFOX | 2 | 4 |
| HIPEC with Oxaliplatin and 5-FU | 2 | 4 |
| Total | 15 | 30 |
| Chemotherapy^†^ |  |  |
| FOLFOX | 13 | 26 |
| FOLFOXIRI | 1 | 2 |
| FOLFIRI and Avastin | 5 | 10 |
| Carboplatin and Paltitaxel | 2 | 4 |
| Untreated | 3 | 6 |
| 5-FU | 13 | 26 |
| 5-FU and Folinic acid | 3 | 6 |
| Oxaliplatin | 2 | 4 |
| Total | 42 | 84 |

*Clinicopathological data were not available for all patients

†Chemotherapeutic data were not available for all patients
